# Comparative Effects of Photobiomodulation Therapy Versus Conventional Antioxidant Supplementation on Serum Glutathione Levels in Euthyroid Chronic Autoimmune Thyroiditis: A Prospective Open-Label Interventional Clinical Trial

**DOI:** 10.3390/antiox15091105

**Published:** 2026-09-01

**Authors:** Venera Berisha-Muharremi, Bernard Tahirbegolli, Alberta Humolli, Reem Hanna

**Affiliations:** 1 Faculty of Medicine, University of Prishtina, Bulevardi I Dëshmorëve nn, 10000 Prishtina, Kosovo; venera.berisha@uni-pr.edu; 2 Endomedica Polyclinic, 84 Muharrem Fejza, Lagjja e Spitalit, 10000 Prishtina, Kosovo; 3 Department of Endocrinology, Clinic of Endocrinology, University Clinical Centre of Kosovo, 10000 Prishtina, Kosovo; 4 Department of Management of Health Institution and Services, Heimerer College, 10000 Prishtina, Kosovo; bernardtahirbegolli@gmail.com; 5 National Sports Medicine Centre, University Clinical Centre of Kosovo, 10000 Prishtina, Kosovo; 6 Faculty of Medicine, University of Ljubljana, 1000 Ljubljana, Slovenia; alberta.humolli@outlook.com; 7 Department of Restorative Dental Sciences, UCL—Eastman Dental Institute, Medical Faculty, University College London, London WC1E 6DE, UK; 8 Head and Neck Academic Centre, Research Department of Integrated Intervention, Division of Surgery and Interventional Science, Medical Faculty, University College London, London W1W 7TY, UK; 9 Department of Surgical Sciences and Integrated Diagnostics, University of Genoa, Viale Benedetto XV, 16132 Genoa, Italy; 10 Dental Faculty, Royal College of Surgeons Ireland (RCSI), 121-122 St Stephen’s Green, Dublin 2, D02 H903 Dublin, Ireland

**Keywords:** antioxidant supplementation, autoimmune thyroiditis, euthyroid state, glutathione, glutathione peroxidases, GSH homeostasis, Hashimoto disease oxidative stress, photobiomodulation therapy, selenium

## Abstract

Background: Chronic autoimmune thyroiditis (CAT) is characterized by persistent autoimmune activity and increased oxidative stress, which may contribute to thyroid follicular injury and disease progression. Photobiomodulation (PBM) has shown potential beneficial effects on thyroid function and autoimmunity; however, its independent effect on systemic antioxidant status, particularly serum glutathione (GSH), has not been established. This study aimed to evaluate the effect of thyroid-directed PBM on serum GSH concentrations and thyroid-related outcomes in treatment-naïve euthyroid women with CAT. Methods: This prospective, non-randomized, open-label, parallel-group comparative interventional study included 50 treatment-naïve euthyroid women with CAT. Participants were allocated to receive either thyroid-directed transdermal PBM (*n* = 25) or selenium plus vitamin D supplementation (*n* = 25). The primary outcome was the change in serum GSH concentration. Secondary outcomes included changes in thyroid-stimulating hormone (TSH), free triiodothyronine (FT3), free thyroxine (FT4), anti-thyroid peroxidase antibodies (anti-TPO), anti-thyroglobulin antibodies (anti-Tg), thyroid volume, and anthropometric parameters. Assessments were performed at baseline (T0), after the intervention period (T1), and three months after intervention completion (T2). Results: A significant time × group interaction was observed for serum GSH concentrations (F = 19.101, *p* < 0.0001). Significant interactions were also observed for anti-TPO (F = 7.513, *p* = 0.001), anti-Tg (F = 7.389, *p* = 0.002), and thyroid volume (F = 13.081, *p* < 0.0001). In the PBM group, GSH increased from baseline to T1 and remained higher at T2, while TSH, anti-TPO, anti-Tg, and thyroid volume decreased, and FT3 and FT4 increased. No significant longitudinal changes in GSH or thyroid autoantibodies were observed in the supplementation group. No participant in the PBM group required levothyroxine (LT4) initiation during follow-up, whereas 24% of participants in the supplementation group initiated LT4 therapy at T1, with some requiring dose escalation by T2. Conclusions: Thyroid-directed PBM was associated with improved systemic GSH status and favorable changes in thyroid autoimmunity, thyroid function parameters, and thyroid volume compared with selenium plus vitamin D supplementation in treatment-naïve euthyroid women with CAT. These findings suggest that modulation of oxidative stress may represent one potential biological pathway underlying the observed effects of PBM. Larger randomized controlled studies with longer follow-up are required to confirm these findings and define the clinical role of PBM in autoimmune thyroid disease.

## 1. Introduction

### 1.1. Chronic Autoimmune Thyroiditis

Chronic autoimmune thyroiditis (CAT), commonly referred to as Hashimoto’s thyroiditis (HT), is the most prevalent organ-specific autoimmune disease [1,2,3,4]. The disease is characterized by a progressive immune-mediated destruction of thyroid tissue resulting from the loss of immunological tolerance to thyroid-specific antigens. Although CAT frequently remains clinically silent during its early stages, persistent autoimmune activity gradually impairs thyroid function, ultimately leading to hypothyroidism in a substantial proportion of affected individuals [1,2].

The pathogenesis of CAT involves a complex interplay between genetic susceptibility, environmental factors, and immune dysregulation. Autoreactive T lymphocytes, B lymphocytes, macrophages, and dendritic cells infiltrate the thyroid gland, initiating a chronic inflammatory response that promotes the production of thyroid-specific autoantibodies, principally thyroid peroxidase antibodies (anti-TPO) and anti-thyroglobulin antibodies (anti-Tg). Sustained immune activation induces apoptosis of thyroid follicular cells, progressive destruction of functional thyroid parenchyma, and gradual replacement of normal tissue with fibrosis, resulting in declining thyroid hormone synthesis and secretion [1,2,3,4].

Current management of CAT is primarily directed toward correcting thyroid hormone deficiency rather than modifying the underlying autoimmune process. Levothyroxine (LT4) replacement therapy remains the standard treatment for patients who develop hypothyroidism and is highly effective in restoring circulating thyroid hormone concentrations and achieving biochemical euthyroidism. However, levothyroxine (LT4) does not directly influence the chronic inflammatory response, oxidative stress (OS), or immune dysregulation responsible for progressive thyroid injury [3,4]. Consequently, many patients continue to exhibit persistent thyroid autoimmunity despite adequate hormone replacement, indicating that normalization of thyroid function does not necessarily reflect resolution of the underlying disease process.

Growing evidence suggests that the euthyroid stage of CAT represents a critical therapeutic window during which interventions targeting disease mechanisms, rather than hormone replacement alone, may help preserve thyroid structure and function [4].

Among the biological processes implicated in disease progression, OS has emerged as a central contributor to both autoimmune-mediated tissue injury and chronic inflammation. Consequently, therapeutic strategies capable of modulating oxidative stress and restoring redox homeostasis have attracted increasing attention as potential adjunctive approaches for the management of CAT [5].

### 1.2. Oxidative Stress (OS) in CAT

Oxidative stress (OS) is increasingly recognized as an important contributor to the pathogenesis and progression of CAT [5]. Unlike most endocrine organs, the thyroid gland operates under inherently oxidative physiological conditions because thyroid hormone biosynthesis requires the continuous generation of reactive oxygen species (ROS), particularly hydrogen peroxide (H_2_O_2_), which serves as an essential substrate for thyroid peroxidase during the iodination and coupling reactions involved in thyroid hormone synthesis [5].

Under physiological conditions, ROS production is tightly regulated by an elaborate intracellular antioxidant network that maintains redox homeostasis and protects thyroid follicular cells from oxidative injury. The thyroid is therefore particularly dependent on an effective antioxidant defense system, including selenium-dependent selenoproteins, which contribute to the detoxification of H_2_O_2_ and other ROS generated during thyroid hormone synthesis [6].

Selenium intake varies considerably across geographic regions, largely depending on the selenium content of soil and food sources, and selenium-dependent enzymes play an important role in maintaining antioxidant protection and thyroid redox homeostasis [7,8].

In CAT, this finely balanced redox system becomes disrupted as a consequence of persistent autoimmune inflammation. Progressive infiltration of the thyroid gland by autoreactive T lymphocytes, macrophages, and other immune cells results in excessive production of ROS and reactive nitrogen species (RNS), exceeding the antioxidant capacity of the tissue [5]. The oxidative imbalance may promote lipid peroxidation, protein oxidation, mitochondrial dysfunction, and oxidative DNA damage, thereby contributing to thyroid follicular cell apoptosis and progressive tissue injury. This chronic oxidative environment may also amplify autoimmune response, creating a self-perpetuating cycle in which inflammation and oxidative stress reinforce one another.

At the molecular level, excessive ROS activate multiple redox-sensitive signaling pathways involved in immune regulation and inflammatory responses. Among these, activation of nuclear factor kappa B (NF-κB) plays a pivotal role by promoting transcription of numerous pro-inflammatory mediators, including tumor necrosis factor-α (TNF-α), interleukin (IL)-1β, IL-6, and interferon-γ (IFN-γ), thereby sustaining immune cell recruitment and perpetuating chronic thyroid inflammation [5]. Conversely, impairment of endogenous antioxidant signaling may reduce the activity of cytoprotective pathways responsible for maintaining cellular redox balance, further increasing the susceptibility of thyroid tissue to oxidative injury.

Recognition of OS as a fundamental component of CAT pathophysiology has stimulated interest in therapeutic strategies aimed at restoring redox homeostasis rather than simply correcting thyroid hormone deficiency. In recent years, photobiomodulation (PBM) has emerged as a promising non-pharmacological intervention capable of modulating mitochondrial function and intracellular redox signaling.

Experimental studies indicate that red and near-infrared light are absorbed primarily by mitochondrial cytochrome *c* oxidase (CCO), the terminal enzyme of the electron transport chain, resulting in enhanced mitochondrial respiration, increased ATP synthesis, transient modulation of physiological ROS signaling, and photodissociation of inhibitory nitric oxide from CCO [8,9,10,11,12,13,14]. Rather than suppressing ROS production, PBM appears to restore physiological redox signaling by activating endogenous antioxidant defense mechanisms, including nuclear factor erythroid 2-related factor 2 (Nrf2), while simultaneously attenuating excessive NF-κB activation and chronic inflammatory responses [8,9,10,11,12,13,14,15].

Collectively, these biological effects provide a strong mechanistic rationale for investigating PBM as a therapeutic approach capable of targeting the oxidative and inflammatory pathways underlying CAT.

### 1.3. Glutathione and Redox Homeostasis

Glutathione (GSH) is the most abundant intracellular non-protein thiol and represents one of the principal components of the endogenous antioxidant defense system [16]. Present predominantly in its reduced form, GSH plays a fundamental role in maintaining cellular redox homeostasis by directly scavenging reactive oxygen and nitrogen species and serving as the major substrate for glutathione peroxidases (GPxs), a family of selenium-dependent enzymes that detoxify hydrogen peroxide and lipid hydroperoxides generated during normal cellular metabolism [16]. Beyond its antioxidant function, GSH is essential for preserving mitochondrial integrity; protecting proteins, membrane lipids, and DNA from oxidative damage; regulating apoptosis; and maintaining normal immune cell function through redox-sensitive signaling pathways [16].

Under physiological conditions, intracellular GSH is continuously regenerated from its oxidized form (GSH, disulfide; GSSG) by glutathione reductase, thereby maintaining a high GSH/GSSG ratio that is critical for normal cellular function and mitochondrial bioenergetics [16]. However, persistent oxidative stress associated with CAT increases GSH utilization and may impair its regeneration, resulting in depletion of intracellular antioxidant reserves and disruption of redox homeostasis [5,16]. Reduced GSH availability has been associated with mitochondrial dysfunction, enhanced inflammatory signaling, and increased susceptibility of thyroid follicular cells to oxidative injury, processes that may contribute to the progression of autoimmune-mediated thyroid damage [5,16].

Growing evidence indicates that modulation of glutathione-dependent pathways represents an important therapeutic target in disorders characterized by chronic oxidative stress. Selenium is an essential structural component of GPxs and thioredoxin reductases, enzymes that play a central role in GSH-dependent antioxidant defense by reducing hydrogen peroxide generated during thyroid hormone synthesis [17,18,19]. Vitamin D has also been implicated in cellular redox regulation through activation of Nrf2-dependent antioxidant pathways, which have been associated with enhanced glutathione synthesis and improved antioxidant capacity [20]. Although these interventions may support endogenous antioxidant defenses, their clinical efficacy in restoring systemic redox homeostasis in patients with CAT remains inconsistent.

Given its central role in antioxidant defense, mitochondrial function, and immune regulation, circulating GSH has emerged as a clinically relevant biomarker of systemic redox status and treatment response in diseases associated with OS. Experimental evidence suggests that infrared and low-power laser irradiation may modulate the glutathione antioxidant system [21]. Nevertheless, despite increasing interest in antioxidant-based therapies for CAT, little is known about the isolated effects of thyroid-directed photobiomodulation on systemic glutathione homeostasis. Clarifying whether PBM can modulate circulating GSH concentrations may provide important mechanistic insight into its potential therapeutic role in autoimmune thyroid disease.

### 1.4. Current Therapeutic Approaches

#### 1.4.1. Levothyroxine Therapy

Current treatment of CAT is primarily focused on replacing hormones lost due to defective thyroid function and not directly modifying the autoimmune process that underlies it. LT4 replacement therapy remains the standard treatment for patients who develop hypothyroidism; however, LT4 does not directly impact chronic inflammation, oxidative stress or progressive destruction of thyroid tissue [3,22,23]. As a result, there has been an increasing amount of attention devoted to adjunctive interventions that can modify the pathophysiology of this disease, particularly during the euthyroid stage of CAT. However, the clinical heterogeneity of CAT remains an important challenge when evaluating the efficacy of adjunctive interventions, particularly in small clinical studies.

#### 1.4.2. Selenium Supplementation

Selenium has been extensively studied due to its important function in thyroid antioxidant defenses. It is part of both glutathione peroxides and thioredoxin reductases, which are enzymes that detoxify H_2_O_2_ produced during the process of producing thyroid hormones, thus reducing oxidative damage to thyroid follicular cells [17,18,19]. Studies have shown that selenium supplements can reduce levels of thyroid autoantibodies as well as enhance the antioxidant status; however, there is significant variability with regard to clinical outcome improvement [17,18,19].

#### 1.4.3. Vitamin D Supplementation

Vitamin D is now being investigated as an adjunctive treatment for CAT due to its effects on modulating inflammation and acting as an antioxidant. Experimental data shows that the administration of vitamin D inhibits the production of pro-inflammatory cytokines, induces the differentiation of regulatory t-cells and enhances oxidative defenses by activating Nrf2-dependent signaling pathways and increasing the rate of glutathione synthesis [20]. However, the results of clinical trials investigating the use of supplemental vitamin D in patients with CAT are highly variable, and therefore its overall therapeutic benefit remains uncertain [24,25,26].

#### 1.4.4. Photobiomodulation (PBM) as a Redox-Modulating Therapy

PBM has emerged as a non-invasive therapeutic approach capable of influencing mitochondrial function, cellular metabolism, and inflammatory regulation through exposure to low-intensity red and near-infrared light. The primary photoreceptor involved in PBM is mitochondrial CCO, which absorbs photons within the red and near-infrared wavelength range. Activation of CCO enhances electron transport chain activity, promotes mitochondrial membrane potential, increases ATP production, and induces transient modulation of intracellular ROS signaling [8,9,10,11,12,13,14]. These physiological ROS signals act as secondary messengers that activate adaptive cellular responses, including stimulation of endogenous antioxidant pathways and enhancement of cellular resilience. Several experimental studies have further suggested that PBM can modulate mitochondrial redox homeostasis and enhance endogenous antioxidant activity, supporting its potential role as a redox-modulating intervention [27,28].

Unlike conventional antioxidant approaches that primarily aim to neutralize reactive species directly, PBM appears to regulate redox signaling by improving mitochondrial efficiency and activating endogenous defense mechanisms. Experimental studies have demonstrated that PBM can stimulate Nrf2-mediated antioxidant responses, increase expression of cytoprotective enzymes, and attenuate excessive activation of inflammatory pathways such as NF-κB [8,9,10,11,12,13,14,15]. Through these mechanisms, PBM may reduce oxidative damage, regulate inflammatory signaling, and promote restoration of cellular redox balance.

The biological relevance of PBM to autoimmune thyroid disease is supported by the unique oxidative requirements of the thyroid gland and the central role of mitochondrial dysfunction and chronic inflammation in CAT progression. Previous clinical investigations have reported beneficial effects of thyroid-directed PBM in patients with HT, including reductions in thyroid autoantibody concentrations, improvements in thyroid function parameters, changes in thyroid morphology, and reduced requirements for thyroid hormone replacement therapy [15,24,25,26,29,30,31]. Furthermore, studies combining PBM with nutritional antioxidant interventions, including selenium and vitamin D supplementation, have suggested potential improvements in thyroid homeostasis and disease-related outcomes [15,26].

Despite these encouraging findings, important knowledge gaps remain. Previous clinical studies have primarily focused on thyroid hormone concentrations, autoantibody levels, ultrasound characteristics, and clinical outcomes, whereas the effects of PBM on systemic antioxidant biomarkers have not been adequately investigated. In particular, the isolated effect of thyroid-directed PBM on GSH concentrations in treatment-naïve euthyroid patients with CAT remains unknown. Understanding whether PBM can influence systemic GSH status may provide important mechanistic insight into how this intervention affects oxidative stress and autoimmune activity in thyroid disease.

### 1.5. Study Rationale and Aim

Although OS is increasingly recognized as a key contributor to the pathogenesis of CAT, therapeutic approaches capable of directly improving redox homeostasis remain insufficiently characterized. PBM has demonstrated potential effects on mitochondrial function, inflammatory regulation, and antioxidant defense pathways; however, its specific impact on systemic GSH status in patients with CAT has not been established.

Furthermore, previous studies evaluating PBM in autoimmune thyroid disease have primarily focused on thyroid function, autoantibody concentrations, and structural changes, while the underlying redox-related mechanisms remain incompletely understood.

The present study was designed to investigate whether thyroid-directed transdermal PBM could improve systemic antioxidant status, assessed by changes in serum GSH concentrations, in treatment-naïve euthyroid women with CAT. To provide a comparative evaluation, the effects of PBM were assessed against conventional antioxidant supplementation consisting of selenium and vitamin D if the level was <40 ng/dL. Therefore, we hypothesized that PBM would enhance GSH availability by modulating mitochondrial function and endogenous antioxidant pathways, thereby improving redox balance in individuals with CAT.

## 2. Materials and Methods

### 2.1. Study Design

This prospective, non-randomized, open-label, parallel-group comparative interventional clinical study was designed to evaluate the effect of thyroid-directed transdermal PBM on systemic oxidative status in euthyroid women with CAT. Transparent Reporting of Evaluations with Nonrandomized Designs (TREND) Checklist is provided in Table S1.

Eligible participants were allocated into two intervention groups. The PBM-treated group received thyroid-directed transdermal PBM as a standalone intervention, whereas the supplementation group received oral selenium combined with vitamin D supplementation without PBM. Because the primary aim was to evaluate the independent biological effects of PBM, only treatment-naïve euthyroid participants without previous CAT-directed therapy were included.

Laboratory analyses were performed by personnel blinded to treatment allocation to minimize measurement bias; participants and the treating endocrinologist were not blinded because of the open-label nature of the interventions. Clinical assessments and thyroid ultrasonography were performed using standardized protocols. All PBM procedures were administered by the same experienced endocrinologist to reduce operator-related variability.

The study was conducted at Endomedica Polyclinic, Prishtina, Kosovo, between May 2023 and May 2024. The study protocol was approved by the Institutional Ethics Committee of Kosovo Medical Chamber (Approval nr 71/2023 dated 4 May 2023) and was conducted according to the principles of the Declaration of Helsinki. The trial was registered at Poliklinika Endomedica, Pristina, Kosovo, under registration number 02/2022, obtained on 25 March 2022. Written informed consent was obtained from all participants before enrollment.

#### 2.1.1. Study Population and PICO Framework

The study population consisted of adult women aged 20–50 years with CAT who were euthyroid and had not previously received treatment for thyroid disease or other chronic medical conditions.

The PICO framework was defined as follows:

- Population (P): Adult females aged 20–50 years diagnosed with CAT according to biochemical and ultrasonographic criteria, with preserved thyroid function and no previous CAT-specific treatment.
- Intervention (I): Thyroid-directed transdermal PBM using an λ 820 nm gallium–aluminum–arsenide (GaAlAs) diode laser.
- Comparison (C): Oral selenium supplementation combined with vitamin D supplementation.
- Outcomes (O): The primary outcome was the change in serum GSH concentration. Secondary outcomes included changes in thyroid autoantibodies markers (anti-TPO and anti-Tg), thyroid function parameters (TSH, FT3, and FT4), thyroid volume, and anthropometric measurements.

#### 2.1.2. Eligibility Criteria

Inclusion Criteria

Participants were eligible if they fulfilled all of the following criteria:

- Female sex and age between 20 and 50 years;
- Diagnosis of CAT confirmed by:
- Elevated serum anti-TPO and/or anti-Tg concentrations (anti-TPO ref. range < 34 IU/mL; anti-tg ref range < 115 IU/mL);
- Thyroid ultrasonographic findings compatible with CAT;
- Euthyroid thyroid function, defined as serum TSH, FT4, and FT3 concentrations within laboratory reference ranges;
- No current indication for LT4 replacement therapy at the first visit according to current clinical practice;
- No previous treatment with LT4, PBM, selenium, vitamin D, or other CAT-specific interventions;
- Willingness to comply with the study protocol and provide written informed consent.

Exclusion Criteria

Participants were excluded if they had any of the following conditions:

- Hypothyroidism requiring LT4 replacement therapy;
- Hyperthyroidism or thyrotoxicosis;
- Graves’ disease;
- Pregnancy or breastfeeding;
- Previous thyroid surgery or radioiodine therapy;
- Thyroid malignancy;
- Thyroid nodules requiring surgical intervention or medical management;
- Congenital thyroid abnormalities;
- Postpartum thyroiditis;
- Autoimmune disease other than CAT;
- History of ionizing irradiation and/or neoplasia involving the cervical region;
- Chronic kidney or liver disease;
- Malignancy;
- Uncontrolled cardiovascular disease;
- Diabetes mellitus;
- Tracheal stenosis;
- Active systemic infection;
- Use of corticosteroids, immunosuppressive drugs, immunomodulators, or medications known to interfere with thyroid hormone synthesis, metabolism or transport.
- Previous exposure to therapeutic PBM involving the cervical region.

#### 2.1.3. Patient Cohort

Following screening according to predefined inclusion and exclusion criteria, eligible participants were enrolled consecutively and assigned to one of two parallel intervention groups according to their individual treatment preference rather than by randomization.

Before enrollment, all participants underwent a detailed clinical history assessment and evaluation for relevant comorbidities. Only patients with isolated CAT were included, while individuals with autoimmune diseases other than CAT were excluded. No new autoimmune conditions were diagnosed during the study follow-up period.

Participants in the PBM-treated group (group 1) received only thyroid-directed transdermal PBM therapy as the sole intervention (*n* = 25). Participants in the supplementation group (group 2) received only oral selenium combined with vitamin D supplementation (*n* = 25).

Unlike previous clinical feasibility studies conducted by our group [13,24], participants included in the present study did not receive LT4 therapy during the study period. This approach allowed evaluation of the independent effects of PBM and antioxidant supplementation in treatment-naïve euthyroid women with CAT without the potential confounding effects of thyroid hormone replacement therapy.

Before initiation of the intervention, baseline demographic, clinical, biochemical, ultrasonographic, and anthropometric data were collected for all participants. The duration of CAT was comparable between the two intervention groups at baseline. All participants were treatment-naïve and had no history of previous selenium, vitamin D, or other antioxidant supplementation before study enrollment. These assessments included age; body mass index (BMI); TSH, FT3, and FT4 concentrations; anti-TPO and anti-Tg concentrations; serum GSH concentration; thyroid ultrasound characteristics; and anthropometric measurements.

All participants received standardized recommendations regarding lifestyle factors, including maintenance of usual physical activity levels, avoidance of new dietary supplements or antioxidant preparations, avoidance of excessive iodine supplementation, and avoidance of major dietary changes during the study period.

Clinical and laboratory evaluations were performed at the following time points:

- T0 (baseline): assessment performed before initiation of PBM or antioxidant supplementation;
- T1 (post-intervention assessment): assessment performed immediately after completion of the three-week PBM treatment protocol or corresponding follow-up period in the supplementation group;
- T2 (three-month follow-up): assessment performed three months after completion of the intervention period.

### 2.2. Intervention Protocols

#### 2.2.1. PBM Therapy Protocol

Participants assigned to the PBM-treated group received thyroid-directed transdermal photobiomodulation using an 820 nm gallium–aluminum–arsenide (GaAlAs) diode laser system (Omega XP, Omega Laser Systems Ltd., Essex, UK).

The treatment protocol was adapted from previously published PBM protocols applied to the thyroid region [13,22,23,24,25,26,27], with minor modifications to maintain the therapeutic dosimetry while addressing the objectives of the present investigation.

Before initiation of PBM treatment, thyroid ultrasonography was performed to identify the anatomical borders of both thyroid lobes.

Eight treatment locations were marked on the skin surface using a sterile surgical marker to ensure consistent positioning of the laser probe during each treatment session.

The locations of irradiated points were 4 over the right thyroid lobe and four over the left thyroid lobe, with approximately 1 cm between each point to ensure consistent laser application during the treatment session.

The laser device delivered continuous-wave (CW) near-infrared (NIR) laser light at a wavelength of 820 nm with an output power of 200 mW. Irradiation was performed using a contact, stationary technique, with the probe positioned perpendicular to the skin surface at an angle of 90°.

Each treatment point received laser irradiation for 20 s, corresponding to an energy density (fluence) of 32 J/cm^2^ per point. Eight irradiation points were treated during each session, resulting in a total irradiation time of 160 s and a total delivered energy density of 256 J/cm^2^ per session.

Participants received a total of six PBM sessions administered twice a week (excluding weekend) over three consecutive weeks, with a minimum interval of 48 h between treatment sessions.

All PBM procedures were performed by the same experienced endocrinologist to minimize operator-dependent variability. The complete PBM treatment parameters are summarized in Table 1.

#### 2.2.2. Selenium and Vitamin D Supplementation Protocol

Participants in the supplementation group received oral antioxidant supplementation consisting of selenium and vitamin D without PBM treatment.

Selenium was administered at a daily dose of 100 μg of elemental selenium in the form of selenomethionine throughout the intervention period.

Vitamin D supplementation was individualized according to baseline serum 25-hydroxyvitamin D [25(OH)D] concentrations. Participants with vitamin D deficiency received replacement supplementation according to their baseline status until adequate serum concentrations were achieved. Participants with baseline 25(OH)D concentrations within the adequate range received maintenance vitamin D supplementation throughout the study period.

For group 2, adherence to supplementation was assessed at each follow-up visit through direct participant interviews and review of treatment records or supplementation diaries.

#### 2.2.3. Standard Patient Recommendations

To minimize potential lifestyle-related confounding factors, all participants received identical standardized recommendations before initiation of the intervention.

Participants were instructed to:

- Maintain their usual level of physical activity throughout the study period;
- Avoid initiating new dietary supplements or antioxidant preparations;
- Avoid excessive intake of iodine-containing supplements;
- Avoid major dietary modifications during the intervention period;
- Maintain adequate hydration;
- Report any newly prescribed medications or newly diagnosed medical conditions during follow-up.

No participant received LT4 therapy at baseline, as only euthyroid individuals without an indication for thyroid hormone replacement were included.

### 2.3. Outcome Measures

#### 2.3.1. Primary Outcome

The primary outcome of this study was the change in serum GSH concentration following intervention. The primary outcome was assessed as the overall longitudinal change across the three predefined assessment time points: baseline (T0), immediately after completion of the intervention (T1), and three-month follow-up (T2).

The primary analysis evaluated the difference in the longitudinal pattern of serum GSH change between the PBM-treated and supplementation groups using the time × group interaction. Within-group and pairwise comparisons were performed as supplementary analyses to characterize the temporal pattern of GSH changes.

The primary outcome and statistical decision rule were defined before data collection. Because serum GSH did not meet parametric assumptions, the primary time × group analysis was performed using a linear mixed-effects model, as described in Section 2.6.

#### 2.3.2. Secondary Outcomes

Secondary outcomes included changes in the following parameters evaluated longitudinally at T0, T1, and T2:

- Thyroid-related biochemical outcomes

The following biochemical parameters were evaluated: TSH, FT4, FT3, anti-TPO, and anti-Tg.

- Thyroid ultrasonographic outcomes

Thyroid ultrasound evaluation was performed to assess structural changes during follow-up. The evaluated parameter was thyroid gland volume.

- Anthropometric outcomes

Anthropometric parameters included: body weight, height, BMI, waist circumference, hip circumference, and WHR.

### 2.4. Assessment Methods

#### 2.4.1. Thyroid Ultrasonography

Thyroid ultrasonography was performed using a high-resolution ultrasound system (GE Logiq V5, GE Healthcare, Düsseldorf, Germany). All ultrasound examinations were conducted by the same experienced endocrinologist throughout the study to minimize inter-observer variability.

Ultrasound evaluation was performed according to established international recommendations for assessment of autoimmune thyroid disease. The thyroid gland volume was evaluated.

The dimensions of each thyroid lobe were measured in longitudinal and transverse planes. The volume of each thyroid lobe was calculated using the ellipsoid formula:

*Volume* (mL) = *Length* × *Width* × *Depth* × 0.523 (1)

The total thyroid volume was calculated as the sum of the volumes of the right and left thyroid lobes. The isthmus was excluded from volume calculations unless it was significantly enlarged.

Changes in thyroid volume and sonographic characteristics were assessed longitudinally at T0, T1, and T2.

#### 2.4.2. Laboratory Measurements

Venous blood samples were collected from each participant after an overnight fast.

All biochemical analyses were performed in the same certified clinical laboratory using standardized laboratory procedures. The serum levels of the following parameters were measured: TSH, FT4, FT3, anti-TPO, anti-Tg, and GSH.

Serum GSH concentrations were quantified using colorimetric assay kit based on the enzymatic DTNB (Ellman’s reagent) recycling method.

Thyroid hormones and thyroid autoantibodies were measured using electrochemiluminescence immunoassay (ECLIA) performed on the Cobas e411 automated analyzer (Roche Hitachi High Technologies Corporation 1-24-14 Nishi-Shinbashi, Minato-ku, Tokyo 105-8717, Japan), according to the manufacturer’s instructions.

#### 2.4.3. Anthropometric Assessment

The following anthropometric measurements were performed at each assessment time point (T0, T1, and T2):

- Body weight was measured to the nearest 0.1 kg using a calibrated digital scale, with participants wearing light clothing without footwear.
- Height was measured to the nearest 0.5 cm using a wall-mounted stadiometer.
- BMI was calculated as:

*BMI* = *weight* (kg)/*height*^2^ (m^2^) (2)

- Waist circumference was measured midway between the lower rib margin and the iliac crest using a standardised measuring tape.
- Hip circumference was measured at the level of the maximum gluteal protuberance.
- The WHR was calculated by dividing waist circumference by hip circumference.

### 2.5. Data Collection

**Figure 1:**
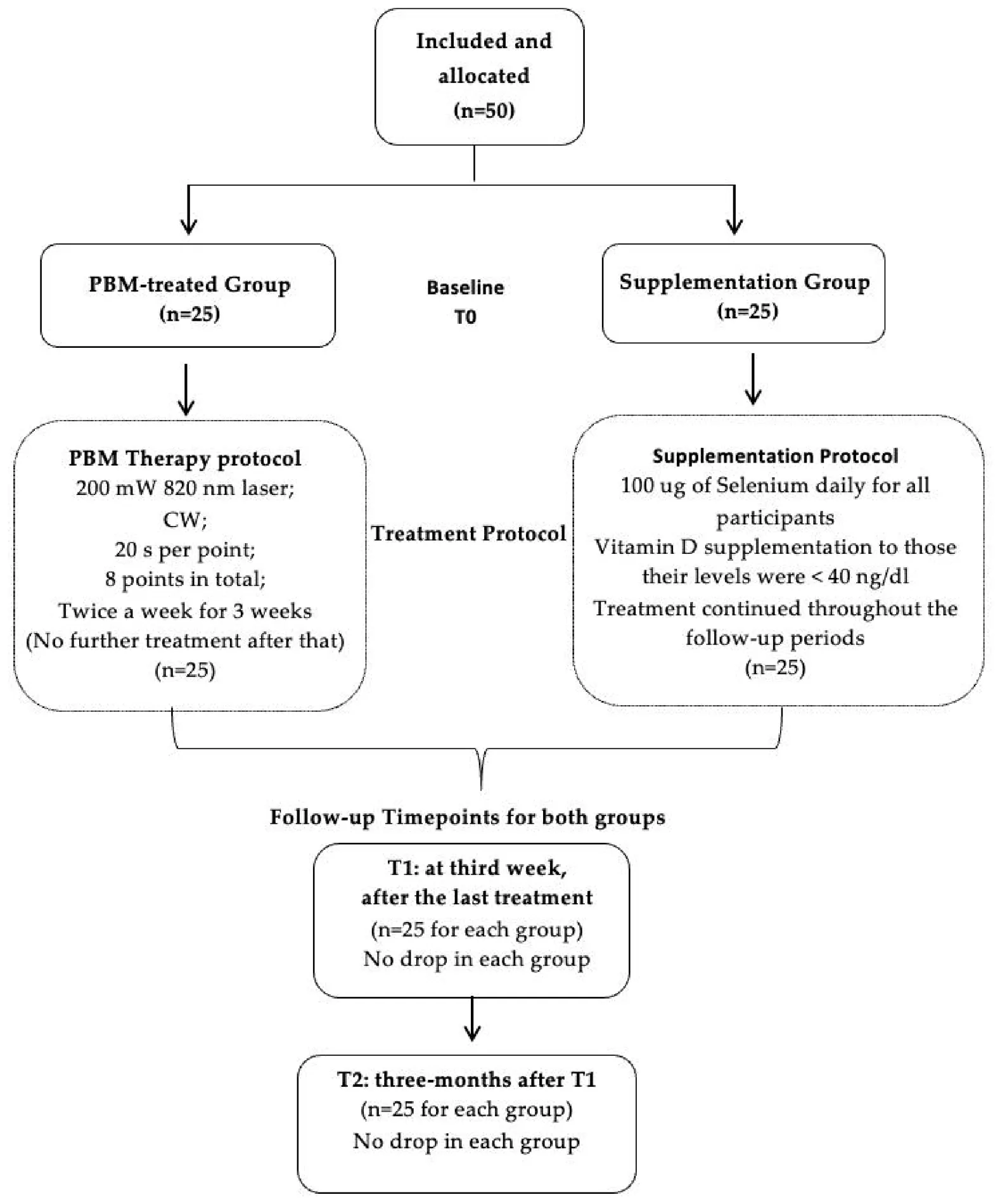
Cohort flowchart showing the study participants.

Clinical, biochemical, ultrasonographic, and anthropometric data were collected at three predefined study time points (T0, T1, and T2) using standardized protocols to ensure consistency. Baseline demographic characteristics, including age and BMI, were recorded. Clinical and biochemical assessments included thyroid function parameters, thyroid autoantibody levels, and serum GSH concentrations. Thyroid ultrasonography was performed to measure thyroid volume. All measurements were obtained at each study time point. Data were entered into a dedicated database and reviewed for completeness and accuracy before statistical analysis. The cohort flowchart is presented in Figure 1.

### 2.6. Statistical Analysis

Statistical analyses were performed using IBM SPSS Statistics for Windows, version 21.0 (IBM Corp., Armonk, NY, USA).

Continuous variables were assessed for distributional characteristics using the Shapiro–Wilk test, supported by graphical evaluation of histograms and Q–Q plots. Normally distributed continuous variables are presented as mean ± standard deviation (SD), whereas non-normally distributed variables are presented as median and interquartile range (IQR). Categorical variables are presented as frequencies (*n*) and percentages (%).

Baseline between-group comparisons were performed using the independent-samples *t*-test for normally distributed variables and the Mann–Whitney U test for non-normally distributed variables.

To evaluate longitudinal changes within the PBM-treated group and the supplementation group (selenium and vitamin D) and to compare temporal changes between groups (time × group interaction), different analytical approaches were applied according to the distributional characteristics of each variable. Repeated measurements analysis of variance (RM-ANOVA) was used for normally distributed variables, while linear mixed-effects models (LMMs) were applied to variables that did not meet parametric assumptions. Within-group longitudinal changes were assessed using RM-ANOVA for normally distributed variables and Friedman’s test for non-normally distributed variables. For the prespecified primary outcome, serum GSH, the primary analysis was the time × group interaction across T0, T1, and T2, with the statistical decision rule defined before data collection.

In the repeated-measures ANOVA, the assumptions of sphericity were assessed using Mauchly’s test, and Greenhouse–Geisser corrections were applied whenever the assumption of sphericity was violated.

Effect sizes were expressed as partial eta squared (η^2^p) or Kendall’s W. When significant main effects of time were identified, Bonferroni-adjusted pairwise comparisons were performed to determine differences between individual time points (T0, T1, and T2). A two-sided *p*-value < 0.05 was considered statistically significant.

As G*Power (version 3.1) does not include a dedicated module for linear mixed-effects models, a sensitivity analysis was performed using the repeated-measures within–between interaction framework as an approximation. Using a significance level of α = 0.05, a desired statistical power of 80%, two study groups, and three repeated assessments, the final sample of 50 participants was sufficient to detect a minimum effect size of Cohen’s f = 0.182.

## 3. Results

### 3.1. Participants Characteristics

A total of 50 women with CAT were included in the study. Twenty-five participants received thyroid-directed PBM (group 1), while 25 participants received selenium combined with vitamin D supplementation (group 2). All participants were female, with an overall mean age of 29.96 ± 4.45 years.

Baseline demographic and clinical characteristics were comparable between the two intervention groups (Table 2).

The mean age was 30.04 ± 4.63 years in the PBM-treated group and 29.88 ± 4.35 years in the supplementation group (selenium and vitamin D).

Baseline BMI values were similar between groups, with mean BMI values of 30.56 ± 3.71 kg/m^2^ in the PBM-treated group and 30.68 ± 3.31 kg/m^2^ in the supplementation group.

The median duration since diagnosis was 12 months (IQR: 4.25–24.00 months) for the total participants in the study.

Baseline demographic and clinical characteristics are summarized in Table 2.

### 3.2. Overall Time × Group Interaction Analysis

To determine whether the trajectory of change differed between the PBM-treated group and the supplementation group, the time × group interaction effect was examined for all outcome variables.

Linear mixed-effects models demonstrated statistically significant time × group interaction effects for TSH (F = 16.760, *p* < 0.0001), anti-TPO (F = 7.513, *p* = 0.001), anti-Tg (F = 7.389, *p* = 0.002), and serum GSH concentrations (F = 19.101, *p* < 0.0001).

Repeated-measures ANOVA demonstrated statistically significant time × group interaction effects for FT4 (F = 10.869, *p* < 0.0001), FT3 (F = 14.897, *p* < 0.0001), thyroid volume (F = 13.081, *p* < 0.0001), BMI (F = 10.421, *p* < 0.0001), and WHR (F = 3.685, *p* = 0.038).

Within-group analysis using repeated-measures ANOVA also demonstrated a statistically significant reduction in WHR from T0 to T1 and T2 in the PBM-treated group (*p* < 0.05), whereas no statistically significant longitudinal change was observed in the supplementation group (*p* > 0.05).

The complete longitudinal analysis of clinical, biochemical, ultrasonographic, and anthropometric parameters is presented in Table 3.

### 3.3. Primary Outcome: Changes in Serum GSH Concentration

Serum GSH concentration was the primary outcome of this study and was evaluated at T0, T1, and T2.

A statistically significant time × group interaction was identified for serum GHS concentrations using linear mixed-effects model analysis (F = 19.101, *p* < 0.0001, η^2^p = 0.443), indicating a different pattern of change over time between the PBM-treated group and the supplementation group (Table 3).

In the PBM-treated group, serum GSH concentrations demonstrated a statistically significant longitudinal increase (χ^2^ = 35.120, *p* < 0.0001, Kendall’s W = 0.702). Median GSH concentration increased from 204.00 mg/L (IQR: 171.50–278.50) at T0 to 255.00 mg/L (IQR: 218.00–299.00) at T1, followed by a decrease to 246.00 mg/L (IQR: 206.50–286.50) at T2.

In contrast, no statistically significant longitudinal change in serum GSH concentration was observed in the supplementation group (χ^2^ = 1.953, *p* = 0.377, Kendall’s W = 0.039). Median GSH concentration changed from 230.00 mg/L (IQR: 176.50–316.50) at baseline (T0) to 211.00 mg/L (IQR: 129.50–286.00) at T1 and 210.00 mg/L (IQR: 136.00–240.00) at T2.

Post hoc pairwise comparisons demonstrated that within the PBM-treated group, GSH concentration was significantly higher at T1 compared with baseline (*p* < 0.05). However, a statistically significant decrease was observed between T1 and T2 (*p* < 0.05). Despite this reduction, GSH concentrations at T2 remained higher than baseline values.

**Figure 2:**
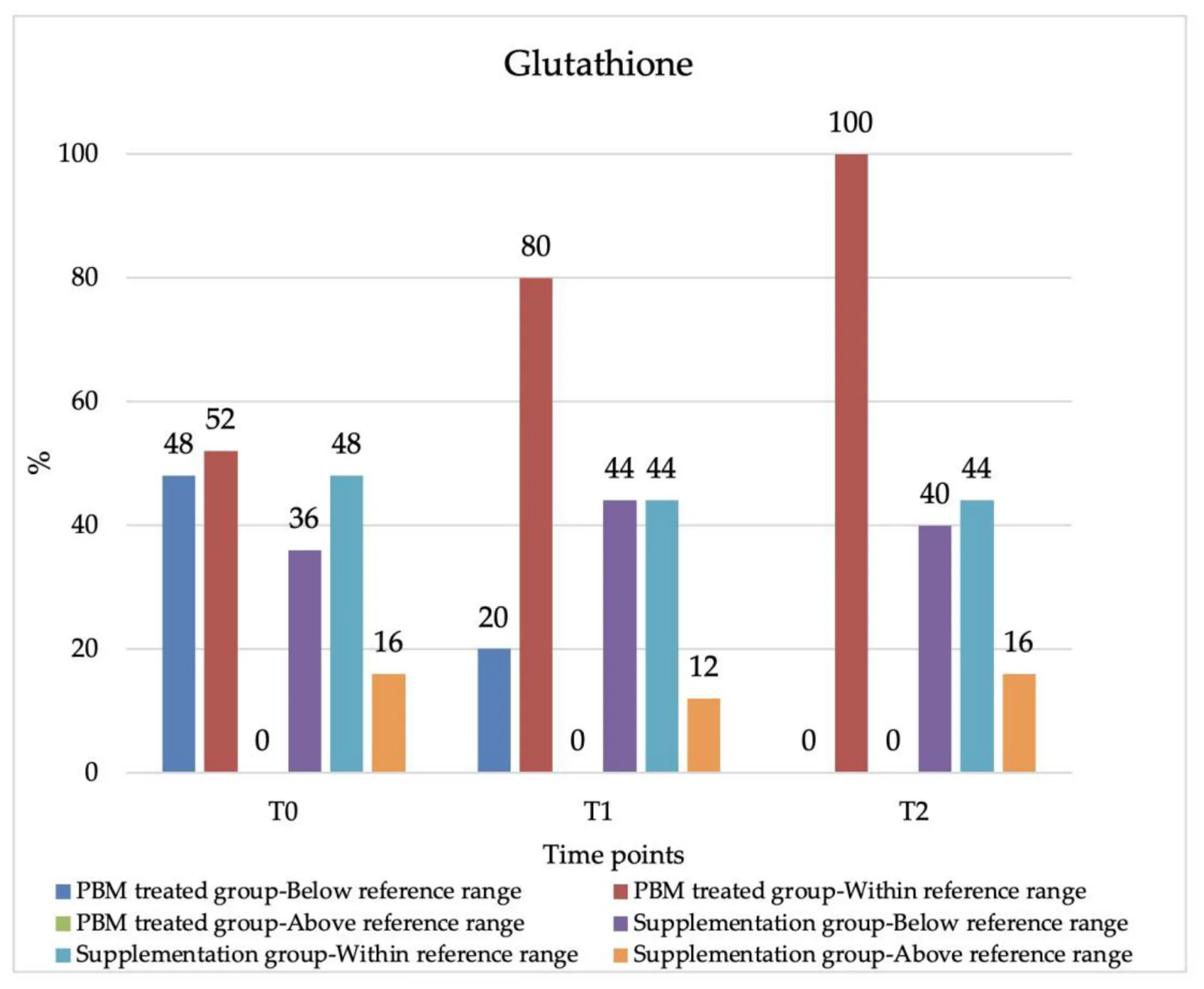
Distribution of participants according to glutathione reference categories over time.

The distribution of participants according to GSH reference categories demonstrated a progressive improvement in the PBM-treated group. The proportion of participants with GSH values within the reference range increased from approximately 52% at baseline to 100% at T2. In contrast, no significant change in GSH reference category distribution was observed in the supplementation group (Figure 2).

### 3.4. Secondary Outcomes

#### 3.4.1. Thyroid Function Parameters

Changes in thyroid function parameters, including TSH, FT4, and FT3 concentrations, were evaluated longitudinally at T0, T1, and T2.

A statistically significant time × group interaction was observed for all evaluated thyroid function parameters. Linear mixed-effects model analysis was used for TSH, whereas repeated-measures ANOVA was used for FT4 and FT3. Linear mixed-effects model analysis demonstrated a significant interaction effect for TSH (F = 16.760, *p* < 0.0001, η^2^p = 0.411). Repeated-measures ANOVA demonstrated significant time × group interaction effects for FT4 (F = 10.869, *p* < 0.0001, η^2^p = 0.185) and FT3 (F = 14.897, *p* < 0.0001, η^2^p = 0.237) (Table 3).

Thyroid-stimulating hormone (TSH)

Within-group analysis demonstrated a statistically significant reduction in serum TSH concentrations over time in the PBM-treated group (χ^2^ = 26.926, *p* < 0.0001, Kendall’s W = 0.538). Median TSH concentration decreased from 2.96 μIU/mL (IQR: 1.55–3.80) at baseline (T0) to 2.50 μIU/mL (IQR: 1.40–2.90) at T1 and 2.20 μIU/mL (IQR: 1.20–2.60) at T2.

In the supplementation group, a statistically significant longitudinal change was also observed (χ^2^ = 7.505, *p* = 0.023, Kendall’s W = 0.150). Median TSH concentration increased from 2.80 μIU/mL (IQR: 1.38–3.80) at baseline to 3.20 μIU/mL (IQR: 2.10–4.20) at T1 and 3.10 μIU/mL (IQR: 2.50–4.40) at T2.

Post hoc pairwise comparisons demonstrated that, in the PBM-treated group, TSH levels were significantly lower at both T1 and T2 compared with baseline (*p* < 0.05). In the supplementation group, TSH levels demonstrated a statistically significant increase between T0 and T1 (*p* < 0.05).

**Figure 3:**
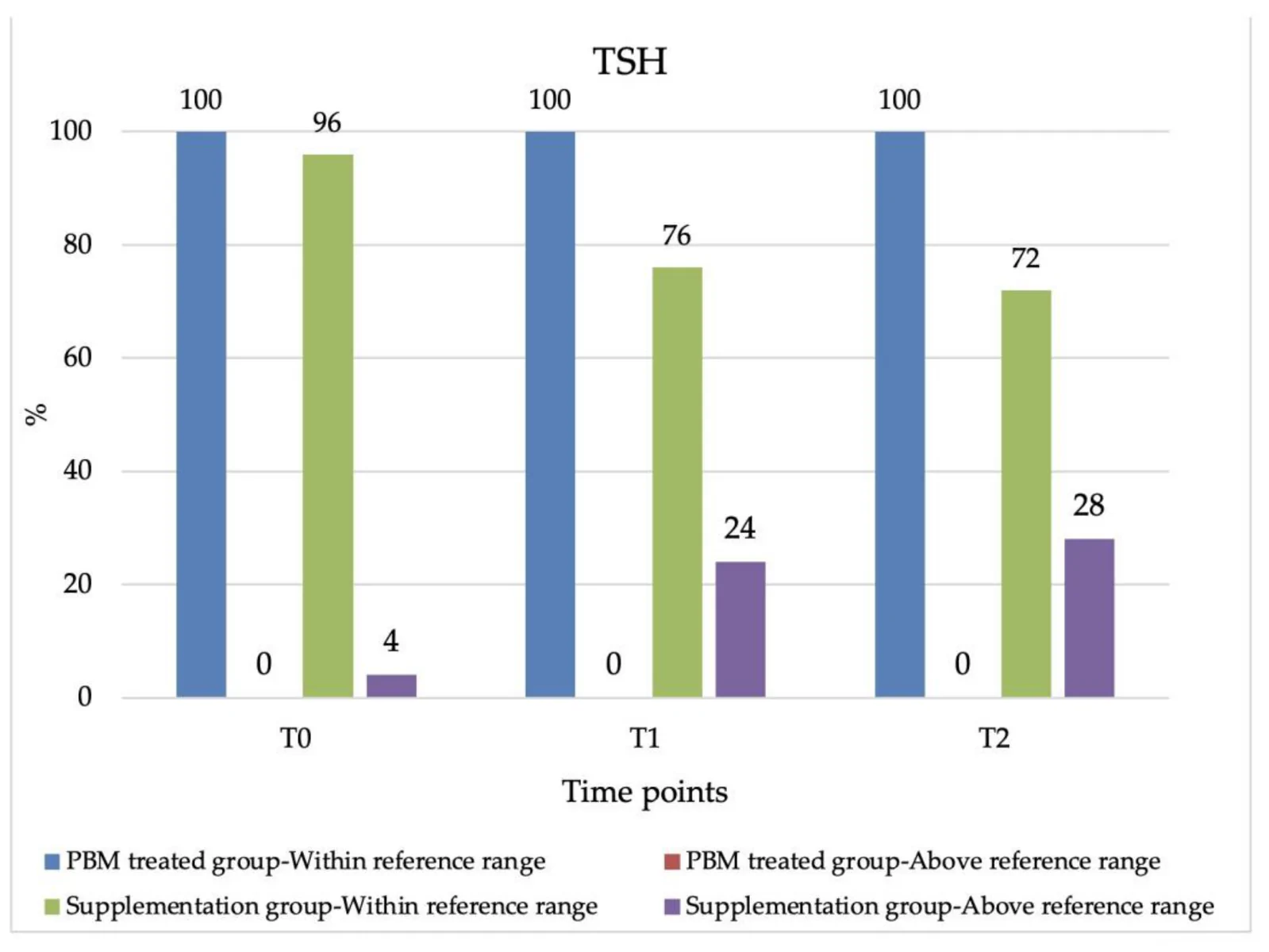
Distribution of participants according to serum TSH reference categories over time.

The proportion of participants with TSH concentrations within the reference range stayed stable at 100% during all study time points in the PBM-treated group. In contrast, the proportion of participants within the reference range declined in the supplementation group from 96% at baseline to 72% at T2 (Figure 3).

Free thyroxine (FT4)

Repeated-measures ANOVA demonstrated a statistically significant increase in FT4 concentrations over time in the PBM-treated group (F = 7.824, *p* = 0.001, η^2^p = 0.246). Mean FT4 concentration increased from 1.42 ± 0.25 ng/dL at baseline to 2.42 ± 1.35 ng/dL at T1 and 2.13 ± 0.96 ng/dL at T2.

In the supplementation group, FT4 concentrations demonstrated a statistically significant longitudinal decrease (F = 4.589, *p* = 0.015, η^2^p = 0.161). Mean FT4 concentration decreased from 1.57 ± 0.39 ng/dL at baseline to 1.36 ± 0.36 ng/dL at T1 and 1.31 ± 0.26 ng/dL at T2 (Table 3).

Post hoc pairwise comparisons demonstrated that FT4 concentrations were significantly higher at T1 and T2 compared with baseline in the PBM-treated group (*p* < 0.05). In contrast, the supplementation group demonstrated a significant decrease in FT4 from T0 to T2 (*p* < 0.05).

**Figure 4:**
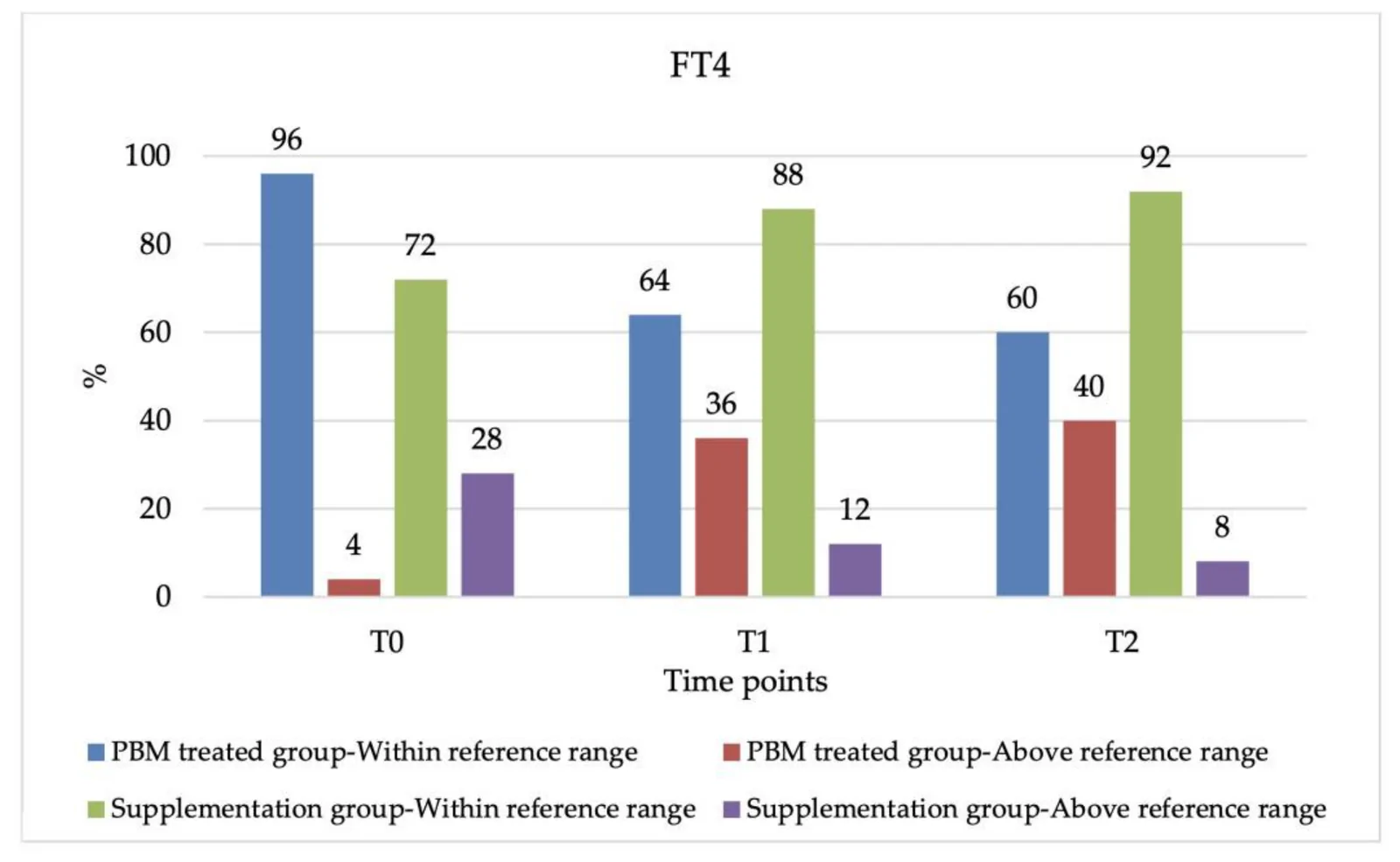
Distribution of participants according to serum FT4 reference categories over time.

The proportion of participants with FT4 concentrations above the reference range increased from 4% at baseline to 36% at T1 and 40% at T2 in the PBM-treated group, whereas the proportion of participants within the reference range declined accordingly. In the supplementation group, the proportion of participants within the reference range increased from 72% at baseline to 92% at T2 (Figure 4).

Free triiodothyronine (FT3)

Repeated-measures ANOVA demonstrated a statistically significant increase in FT3 concentrations over time in the PBM-treated group (F = 10.767, *p* = 0.001, η^2^p = 0.310). Mean FT3 concentration increased from 3.73 ± 1.15 pmol/L at baseline to 5.00 ± 1.34 pmol/L at T1 and 5.08 ± 0.55 pmol/L at T2.

In the supplementation group, FT3 concentrations demonstrated a statistically significant longitudinal decrease (F = 4.178, *p* = 0.021, η^2^p = 0.148).

Mean FT3 concentration decreased from 4.22 ± 1.04 pmol/L at baseline (T0) to 3.80 ± 0.99 pmol/L at T1 and 3.72 ± 0.93 pmol/L at T2.

Bonferroni-adjusted pairwise comparisons demonstrated that FT3 concentrations were significantly higher at T1 and T2 compared with baseline in the PBM-treated group (*p* < 0.05).

**Figure 5:**
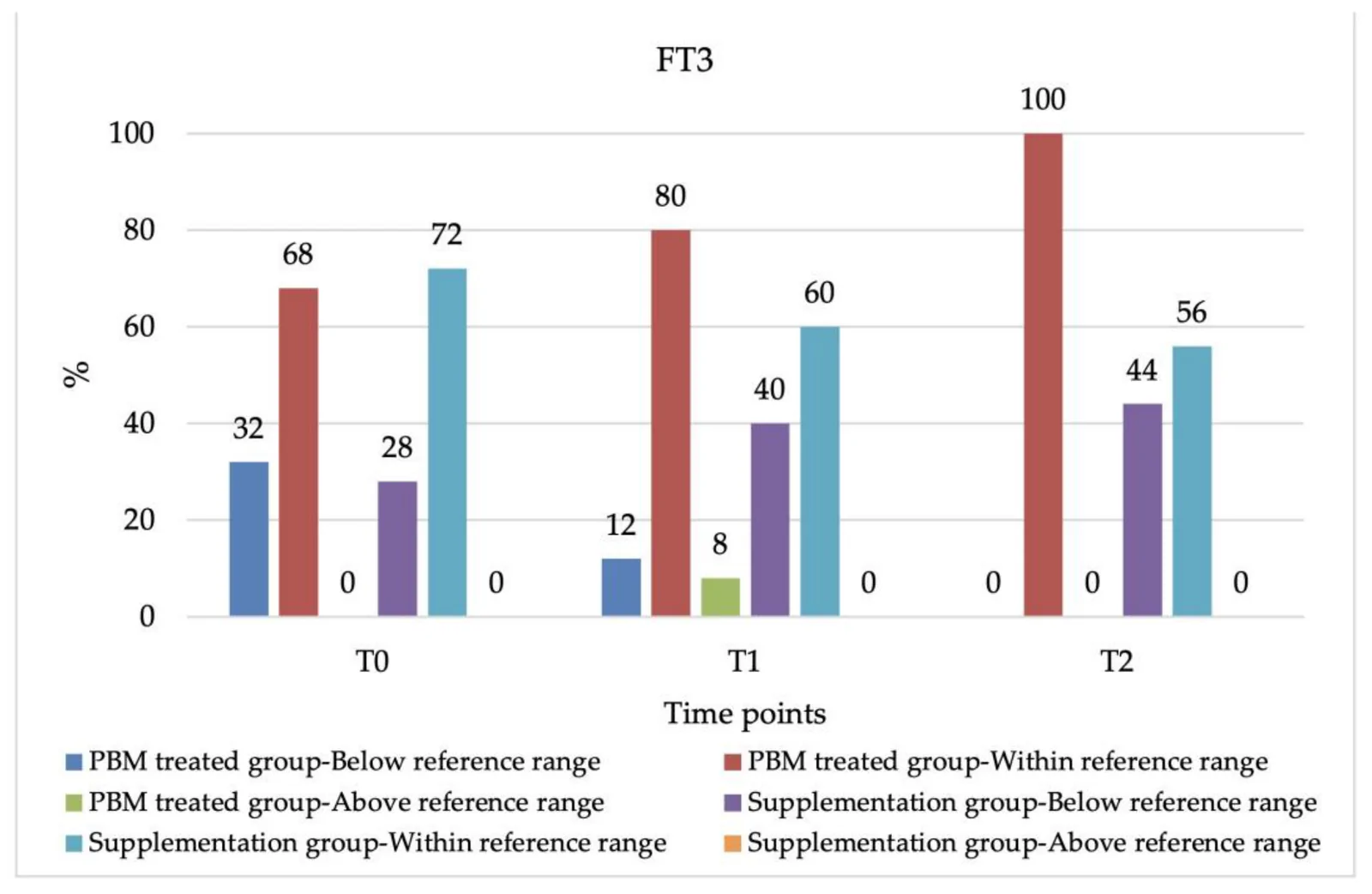
Distribution of participants according to serum FT3 reference categories over time.

The PBM-treated group showed a progressive shift of FT3 concentrations, with the proportion of participants within the reference range increasing from 68% at baseline to 100% at T2. In the supplementation group, the proportion of participants within the reference range increased from 28% at baseline to 44% at T2 (Figure 5).

#### 3.4.2. Thyroid Autoimmunity Markers

Serum thyroid autoantibody concentrations, including anti-TPO and anti-Tg, were evaluated at T0, T1, and T2.

A statistically significant time × group interaction was observed for both thyroid autoantibody parameters, anti-TPO (F = 7.513, *p* = 0.001, η^2^p = 0.238) and anti-Tg (F = 7.389, *p* = 0.002, η^2^p = 0.238) (Table 3).

Anti-thyroid peroxidase antibodies (anti-TPO)

Baseline anti-TPO concentrations were numerically higher in the PBM-treated group [443.00 IU/mL (IQR: 149.55–595.85)] than in the supplementation group [268.50 IU/mL (IQR: 38.50–554.60)]. However, the distributions showed substantial overlap, and the between-group difference was not statistically significant (Mann–Whitney U = 235.500, z = −1.494, *p* = 0.135; rank-biserial correlation = 0.246).

Within-group analysis demonstrated a statistically significant reduction in anti-TPO concentrations over time in the PBM-treated group (χ^2^ = 38.323, *p* < 0.0001, Kendall’s W = 0.766).

In contrast, no statistically significant longitudinal change was observed in the supplementation group (χ^2^ = 3.920, *p* = 0.141, Kendall’s W = 0.078).

Post hoc pairwise comparisons demonstrated that anti-TPO concentrations were significantly lower at both T1 and T2 compared with baseline in the PBM-treated group (*p* < 0.05). No statistically significant changes between time points were observed in the supplementation group (*p* > 0.05).

**Figure 6:**
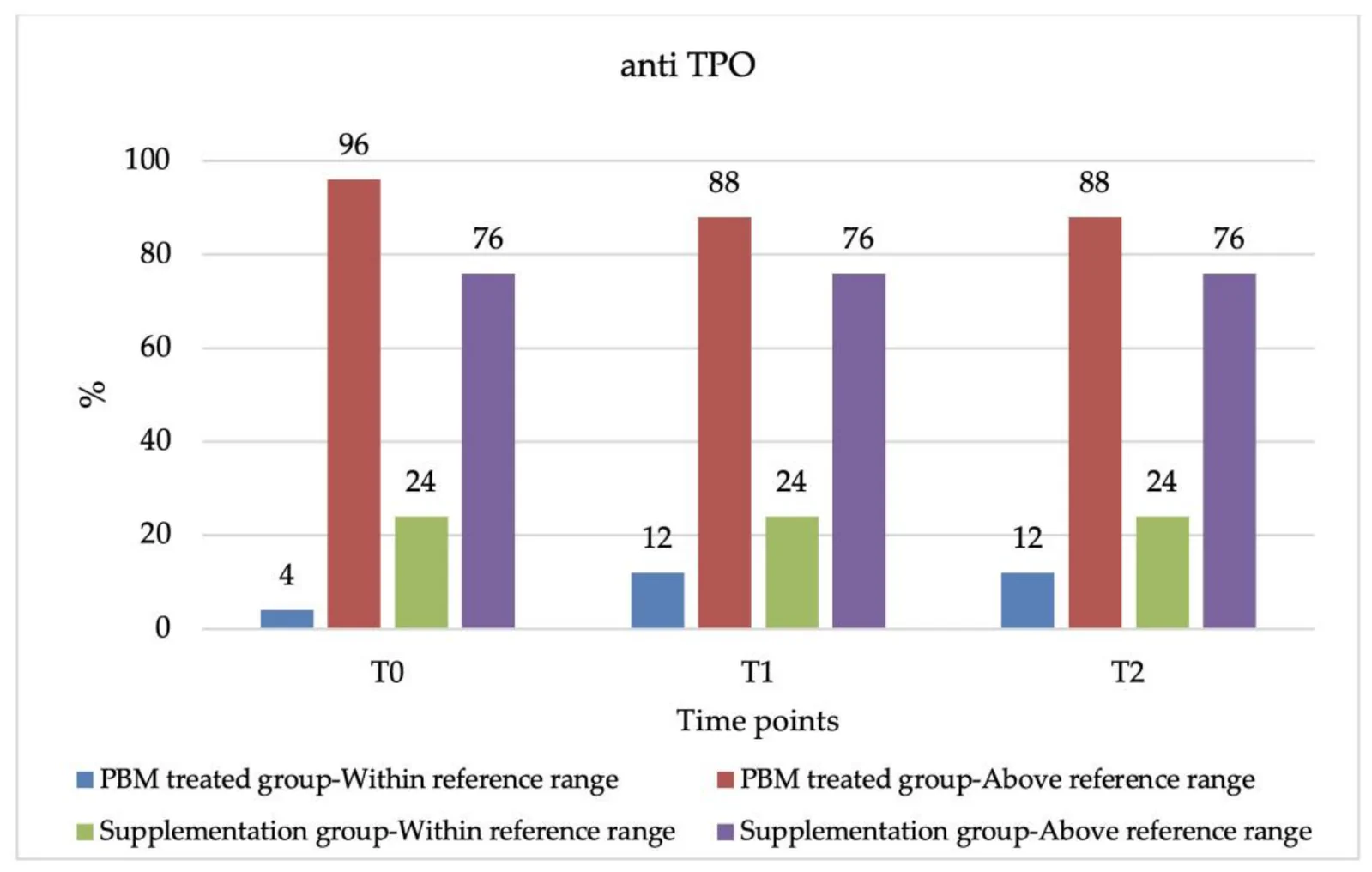
Distribution of participants according to anti-TPO reference categories over time.

The proportion of participants with anti-TPO concentrations within the reference range increased from 4% at baseline to 12% at both follow-up assessments in the PBM-treated group. No changes were observed in the supplementation group, where approximately one-quarter of participants remained within the reference range throughout the study (Figure 6).

Anti-thyroglobulin antibodies (anti-Tg)

Within-group analysis demonstrated a statistically significant reduction in anti-Tg concentrations over time in the PBM-treated group (χ^2^ = 18.589, *p* < 0.0001, Kendall’s W = 0.371). Median anti-Tg concentration decreased from 173.20 IU/mL (IQR: 25.85–420.52) at baseline (T0) to 67.60 IU/mL (IQR: 20.00–205.00) at T1 and 40.00 IU/mL (IQR: 17.50–109.42) at T2.

The supplementation group demonstrated no statistically significant longitudinal change in anti-Tg concentrations (χ^2^ = 0.320, *p* = 0.852, Kendall’s W = 0.006). Median anti-Tg concentration changed from 52.88 IU/mL (IQR: 19.31–236.80) at T0 to 108.70 IU/mL (IQR: 18.65–340.25) at T1 and 97.90 IU/mL (IQR: 18.90–385.60) at T2.

Post hoc pairwise comparisons demonstrated that anti-Tg concentrations were significantly lower at both T1 and T2 compared with baseline in the PBM-treated group (*p* < 0.05). No statistically significant longitudinal changes were observed in the supplementation group (*p* > 0.05).

**Figure 7:**
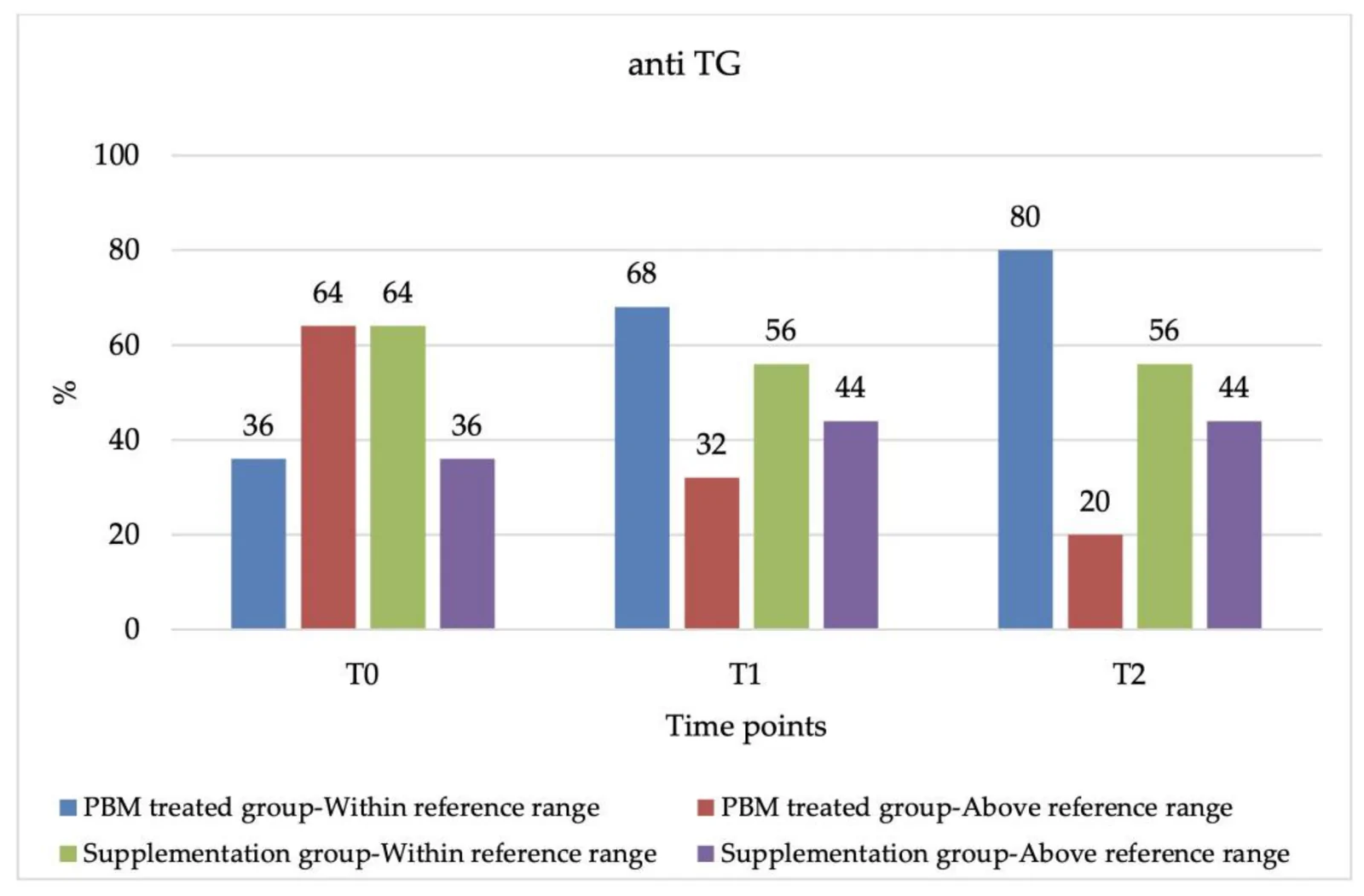
Distribution of participants according to anti-TG reference categories over time.

The distribution of participants according to thyroid autoantibody reference categories further supported the longitudinal findings. In the PBM-treated group, the proportion of participants with anti-TP values within the reference range increased from 36% at baseline to 80% at T2, whereas the distribution remained largely unchanged in the supplementation group (64% at baseline, 56% at T2) (Figure 7).

#### 3.4.3. Thyroid Volume and Ultrasonographic Findings

Thyroid volume was evaluated by ultrasonography at baseline (T0), after completion of the intervention period (T1), and at three-month follow-up (T2). A statistically significant time × group interaction was observed for thyroid volume, indicating different longitudinal patterns between the PBM-treated group and the supplementation group.

Repeated-measures ANOVA demonstrated a statistically significant time × group interaction effect for thyroid volume (F = 13.081, *p* < 0.0001, η^2^p = 0.214) (Table 3).

Within-group analysis demonstrated a statistically significant reduction in thyroid volume in the PBM-treated group (F = 13.646, *p* < 0.0001, η^2^p = 0.362).

Mean thyroid volume decreased from 15.86 ± 3.16 mL at baseline (T0) to 13.56 ± 1.46 mL at T1 and 12.14 ± 3.39 mL at T2.

In contrast, no statistically significant longitudinal change was observed in the supplementation group (F = 0.121, *p* = 0.886, η^2^p = 0.005). Mean thyroid volume remained relatively stable, changing from 15.43 ± 3.13 mL at baseline to 15.37 ± 3.37 mL at T1 and 15.43 ± 3.44 mL at T2.

Post hoc pairwise comparisons analysis demonstrated that thyroid volume in the PBM-treated group was significantly lower at both T1 and T2 compared with baseline (*p* < 0.05). No statistically significant differences between time points were identified in the supplementation group (*p* > 0.05).

**Figure 8:**
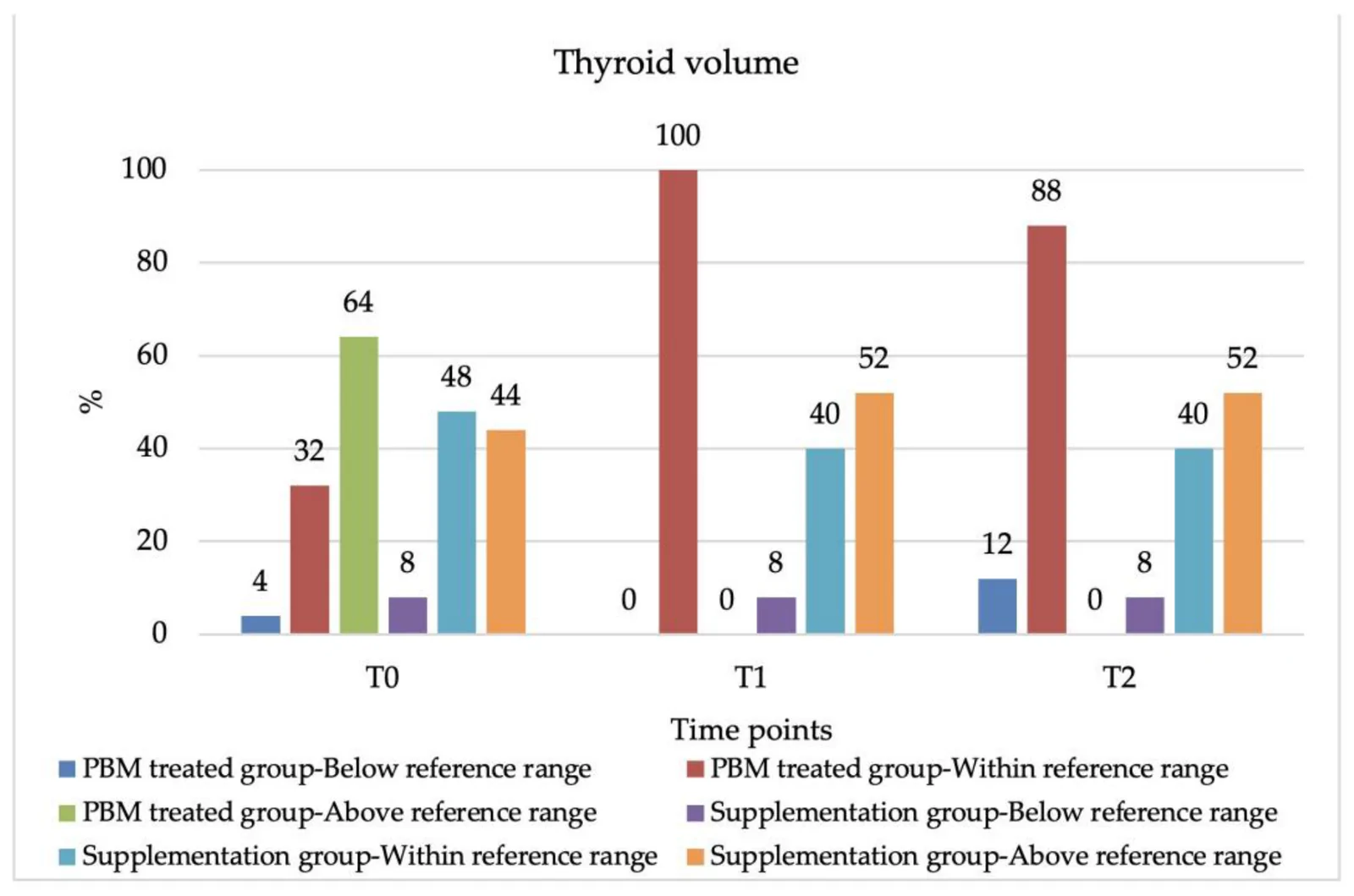
Distribution of participants according to thyroid volume reference categories over time.

A pronounced shift toward normal thyroid volume was observed in the PBM-treated group, with the proportion of participants within the reference range increasing from 32% at baseline to 88% at T2 and no participants remaining above the reference range at follow-up. In the supplementation group, the distribution across thyroid volume categories remained largely unchanged over time (Figure 8).

#### 3.4.4. Anthropometric Outcomes

Anthropometric parameters, including BMI and WHR, were assessed at T0, T, and T2.

A statistically significant time × group interaction was observed for both BMI and WHR. Repeated-measures ANOVA demonstrated a significant interaction effect for BMI (F = 10.421, *p* < 0.0001, η^2^p = 0.693) and WHR (F = 3.685, *p* = 0.038, η^2^p = 0.071) (Table 3).

Body mass index (BMI)

Within-group analysis demonstrated a statistically significant change in BMI over time in the PBM-treated group (F = 11.436, *p* < 0.0001, η^2^p = 0.323).

Mean BMI significantly changed from T0 to post-treatment assessment (T1 and T2) in the PBM-treated group; however, post hoc pairwise comparisons demonstrated that BMI was significantly reduced between T0 and T2 and between T1 and T2 (*p* < 0.05), while no statistically significant difference was observed between T0 and T1.

Mean BMI changed from 30.56 ± 3.71 kg/m^2^ at T0 to 30.54 ± 3.82 kg/m^2^ at T1 and 29.88 ± 3.84 kg/m^2^ at T2.

In contrast, no statistically significant longitudinal change was observed in the supplementation group (F = 1.466, *p* = 0.240, η^2^p = 0.058). Mean BMI remained stable, changing from 30.68 ± 3.31 kg/m^2^ at baseline to 30.84 ± 3.26 kg/m^2^ at T1 and 30.88 ± 3.30 kg/m^2^ at T2.

Waist-to-hip ratio (WHR)

Repeated-measures ANOVA demonstrated a statistically significant time × group interaction effect for WHR (F = 3.685, *p* = 0.038, η^2^p = 0.071).

Within-group analysis demonstrated a statistically significant reduction in WHR over time in the PBM-treated group (F = 6.252, *p* = 0.011, η^2^p = 0.207). Mean WHR decreased from 0.88 ± 0.06 at T0 to 0.88 ± 0.06 at T1 and 0.86 ± 0.06 at T2.

No statistically significant longitudinal change was observed in the supplementation group (F = 0.590, *p* = 0.491, η^2^p = 0.024). Mean WHR remained unchanged from baseline (0.90 ± 0.07) to T1 (0.90 ± 0.07) and T2 (0.90 ± 0.07).

Post hoc pairwise comparisons demonstrated that WHR was significantly lower at T2 compared with T0 in the PBM-treated group (*p* < 0.05).

### 3.5. Clinical Treatment Changes During Follow-Up

At T0, none of the participants in either the PBM-treated group or the supplementation group were receiving LT4 therapy, as all participants were euthyroid and did not meet criteria for thyroid hormone replacement.

**Figure 9:**
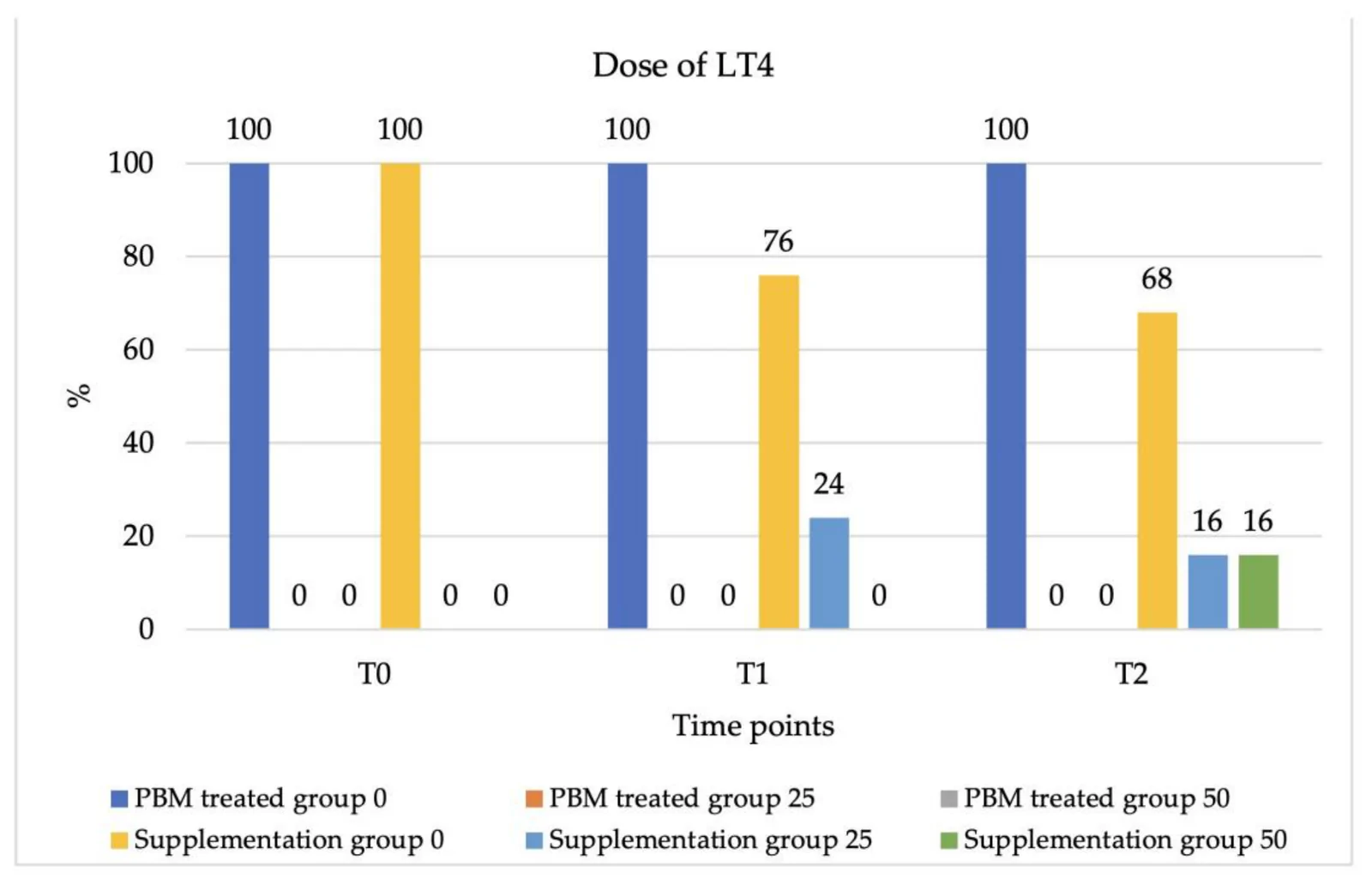
Changes in levothyroxine (LT4) dose during follow-up in the PBM-treated and supplementation groups.

During follow-up (T1 and T2), differences were observed between the two treatment groups regarding the need for initiation of LT4 therapy. In the PBM-treated group, 100% of participants remained free of pharmacological thyroid treatment throughout the follow-up period. In contrast, 24% of participants in the supplementation group initiated LT4 therapy (25 µg/day) at T1. At T2, 16% of participants required dose escalation to 50 µg/day (Figure 9).

No adverse events or unintended effects were observed or reported in either intervention group during the study period.

## 4. Discussion

### 4.1. Principal Findings

The present study found that thyroid-directed PBM was associated with improvements in systemic antioxidant status, thyroid autoimmunity, thyroid morphology, and thyroid function parameters in treatment-naïve euthyroid women with CAT. Among the evaluated outcomes, the increase in serum GSH, the study’s primary endpoint, is particularly noteworthy because oxidative stress is increasingly recognized as an important contributor to thyroid follicular injury and autoimmune progression in CAT.

Importantly, these findings should be interpreted within the context of the study design. The prospective comparative design and evaluation of PBM as an isolated intervention provide valuable preliminary evidence; however, confirmation through larger randomized controlled studies with longer follow-up periods is required before definitive conclusions regarding clinical efficacy can be established.

### 4.2. Effect of PBM on Serum GSH

The primary finding of this study was the significant improvement in serum GSH concentration following thyroid-directed PBM compared with selenium plus vitamin D supplementation. Participants receiving PBM demonstrated a significant longitudinal increase in GSH concentration, whereas no significant change was observed in the supplementation group. These findings suggest that PBM may enhance systemic antioxidant capacity through mechanisms extending beyond direct antioxidant supplementation. However, serum GSH represents one component of the antioxidant defense system and should not be interpreted as a complete measure of total antioxidant capacity. Selenium contributes to antioxidant defense primarily through its incorporation into selenoproteins, including glutathione peroxidases and thioredoxin reductases [32,33], whereas PBM is proposed to influence mitochondrial bioenergetics, physiological ROS signaling, and endogenous antioxidant pathways [8,9,10,11,12,13,14,34]. These different biological mechanisms may partly explain the contrasting GSH responses observed between groups.

The observed response is biologically plausible based on the known mechanisms of PBM. Through interaction with mitochondrial photoreceptors, particularly CCO, PBM may influence mitochondrial respiration, ATP production, mitochondrial membrane potential, and redox signaling pathways [8,9,10,11,12,13,14,34]. Rather than completely suppressing ROS production, PBM appears to regulate redox homeostasis by maintaining physiological ROS signaling while limiting excessive OS. This mechanism may be particularly relevant in CAT, where chronic OS contributes to thyroid follicular injury, mitochondrial dysfunction, and inflammatory activation [5,16].

To our knowledge, this is the first clinical study evaluating changes in serum GSH concentrations following thyroid-directed PBM as a standalone intervention in treatment-naïve euthyroid women with CAT.

The relationship between PBM and GSH metabolism has also been demonstrated experimentally. Kao et al. [21] reported that laser exposure increased GSH levels and enhanced GSH-related enzyme activity in rat hepatocytes, suggesting that PBM may promote endogenous antioxidant defense mechanisms.

Previous clinical studies have also reported reductions in oxidative stress following PBM in patients with HT, supporting a potential role for redox modulation in its therapeutic effects [25,35].

An additional observation was the temporal pattern of the GSH response. Serum GSH concentration increased significantly immediately after PBM treatment and decreased partially during the three-month follow-up period. Nevertheless, GSH concentrations at T2 remained higher than baseline, and all participants were within the reference range. This finding suggests that PBM may induce a sustained but partially attenuated improvement in antioxidant status after completion of treatment. Whether repeated maintenance sessions are required to preserve long-term improvements in GSH homeostasis remains unknown and should be evaluated in future studies.

Future prospective studies should investigate optimal treatment schedules, duration of effect, and whether continued PBM exposure can maintain or further improve antioxidant status in patients with CAT.

### 4.3. Effect of PBM on Thyroid Autoimmunity

An important secondary finding of this study was the significant reduction in thyroid autoantibody concentrations following PBM treatment. Both anti-TPO and anti-Tg levels decreased significantly over time in the PBM-treated group, whereas no significant longitudinal changes were observed in the supplementation group. These findings suggest that PBM may influence the autoimmune component of CAT in addition to improving systemic antioxidant status.

Although baseline anti-TPO concentrations did not differ statistically between the groups, the median value was numerically higher in the PBM-treated group. Given the modest sample size and the considerable within-group variability, a potential contribution of baseline imbalance and regression to the mean cannot be entirely excluded. Therefore, the anti-TPO findings should be interpreted cautiously and confirmed in larger randomized studies.

The reduction in thyroid autoantibody concentrations observed in this study is consistent with previous clinical investigations of PBM in autoimmune thyroid disease. Höfling et al. [24,31] reported reductions in thyroid autoantibodies following low-level laser therapy. Other clinical studies have also demonstrated improvements in thyroid-related outcomes following PBM, either alone or combined with selenium and vitamin D supplementation [15,25,26,36].

The immunomodulatory effects of PBM may involve interactions between mitochondrial function, redox signaling, and inflammatory pathways [8,9,10,11,12,13,14,34]. In CAT, excessive ROS production may contribute to inflammatory activation and thyroid follicular injury. PBM may therefore attenuate excessive inflammatory signaling by modulating mitochondrial function and redox homeostasis.

Additional evidence supporting an immunomodulatory role for PBM comes from Höfling et al. [37], who reported alterations in circulating transforming growth factor-β1 (TGF-β1) following PBM treatment, suggesting possible effects on immune regulation in autoimmune thyroid disease. Although inflammatory cytokines and immune cell populations were not evaluated in the present study, the concurrent improvement in GSH status and reduction in thyroid autoantibodies support a possible role for redox modulation in PBM-associated immune effects.

An interesting observation was the different response pattern between anti-TPO and anti-Tg antibodies. Anti-Tg demonstrated a particularly pronounced improvement, with the proportion of participants within the reference range increasing from 36% at baseline to 80% at T2. Anti-TPO concentrations also decreased substantially, although normalization occurred in fewer participants. The different response patterns between anti-TPO and anti-Tg may reflect differences in antibody kinetics or biological responsiveness; however, these findings require confirmation in larger cohorts.

Overall, these findings suggest that PBM may influence both oxidative and autoimmune components of CAT. The simultaneous improvement in GSH status and reduction in thyroid autoantibody concentrations supports the hypothesis that restoration of redox balance may contribute to reduced autoimmune activity. Future studies incorporating inflammatory cytokine profiling, immune cell characterization, and additional oxidative biomarkers are required to clarify the mechanisms involved.

### 4.4. Effect of PBM on Thyroid Function

In addition to improving systemic antioxidant status and reducing thyroid autoimmunity markers, PBM treatment was associated with favorable changes in thyroid biochemical parameters. Participants receiving PBM demonstrated a significant reduction in serum TSH accompanied by increases in FT4 and FT3 concentrations, whereas the supplementation group showed an increase in TSH and reductions in FT4 and FT3 during follow-up.

The current results are consistent with previous clinical studies investigating the effects of PBM in patients with HT. Höfling et al. [24,31] reported improvements in thyroid hormone production and reduced the subsequent LT4 requirements following low-level laser therapy. Similarly, studies evaluating PBM combined with selenium and vitamin D supplementation demonstrated improvements in thyroid homeostasis during six- and twelve-month follow-up periods [15,26].

The mechanisms underlying these effects remain incompletely understood but may involve interactions between oxidative stress regulation, mitochondrial function, and inflammatory activity. By improving mitochondrial function and regulating redox signaling, PBM may contribute to preservation of thyroid follicular activity and hormone production capacity [8,9,10,11,12,13,14,34].

No participant in the PBM group required LT4 during follow-up, whereas 24% of participants in the supplementation group initiated LT4 at T1 and some required dose escalation by T2. These findings should be interpreted cautiously given the small sample size and non-randomized design. Larger randomized studies with longer follow-up are needed to determine whether PBM influences the long-term requirement for thyroid hormone replacement.

### 4.5. Effect of PBM on Thyroid Volume

The present study demonstrated a significant reduction in thyroid volume following thyroid-directed PBM, with progressive normalization of thyroid size during follow-up. In contrast, no significant longitudinal change in thyroid volume was observed in the supplementation group. These findings suggest that PBM may influence structural characteristics of the thyroid gland in women with CAT.

The reduction in thyroid volume observed following PBM is consistent with previous clinical investigations reporting structural improvements after PBM therapy in autoimmune thyroid disease. Höfling et al. [24,31] demonstrated reductions in thyroid volume following PBM therapy. Similarly, previous studies from our group reported progressive normalization of thyroid volume following PBM during longer follow-up periods [15,26].

The mechanisms underlying changes in thyroid volume remain incompletely understood. In CAT, thyroid enlargement may reflect inflammatory infiltration, immune activation, edema, and structural remodeling associated with chronic autoimmune injury. Therefore, the observed reduction in thyroid volume after PBM may reflect decreased inflammatory activity and improved tissue homeostasis rather than direct structural regeneration. The concurrent improvement in GSH status and reduction in thyroid autoantibody concentrations observed in this study support the possibility that modulation of oxidative and inflammatory pathways contributes to these structural changes.

Although these findings support a potential structural benefit of PBM, additional studies are required to determine whether the reduction in thyroid volume reflects decreased inflammatory activity, reduced tissue edema, or other mechanisms associated with improved thyroid homeostasis.

### 4.6. Clinical Implications

Because PBM is non-invasive, it may represent a potential adjunctive approach during the early stages of autoimmune thyroid disease, particularly in euthyroid patients who do not yet require LT4 therapy. However, it should not currently be considered a replacement for established thyroid hormone replacement therapy or standard clinical management. Larger controlled trials are required before clinical recommendations can be established.

Clinical translation will also require standardization of PBM parameters, including wavelength, power, fluence, treatment duration, and anatomical application site, as biological effects are highly dependent on dosimetric variables [38,39]. Further research should establish optimal thyroid-specific dosimetry and standardized treatment protocols.

Overall, PBM represents a promising investigational approach for modulation of oxidative stress and autoimmune activity in CAT, but confirmation through large randomized controlled trials with longer follow-up and mechanistic biomarkers is required.

### 4.7. Strengths and Limitations

This study has several strengths. The prospective design, inclusion of treatment-naïve euthyroid women with CAT, and evaluation of thyroid-directed PBM as a standalone intervention allowed assessment of the biological effects of PBM without the potential confounding influence of previous thyroid hormone replacement or other CAT-directed therapies. In addition, the evaluation of serum GSH as the primary outcome, together with thyroid function parameters, thyroid autoantibodies, thyroid volume, and anthropometric measures, provided a comprehensive assessment of the potential effects of PBM.

Several limitations should also be acknowledged. The study was conducted at a single center and included a relatively small sample size consisting exclusively of women, which may limit the generalizability of the findings. The relatively small sample size may also have limited the statistical power to adequately assess changes in thyroid autoantibody levels, particularly anti-TPO and anti-Tg, which showed substantial inter-individual variability. Ultrasonographic assessment of thyroid volume may be subject to inherent measurement variability, even when performed by an experienced operator. Furthermore, the non-randomized, preference-based allocation may have introduced selection bias and limits causal inference; therefore, between-group differences should be interpreted cautiously. The open-label design may also have introduced performance bias. The follow-up period was limited to three months after completion of the intervention; therefore, the long-term durability of the clinical and biochemical changes remains uncertain. Potential influences of hormonal status, psychosocial stress, and physical activity on CAT activity were not systematically assessed and may have contributed to disease heterogeneity. Finally, although GSH was selected as the primary marker of oxidative status, additional biomarkers, including glutathione peroxidase activity, oxidized glutathione (GSSG), inflammatory cytokines, and molecular markers of redox regulation, were not assessed and should be incorporated into future mechanistic studies. Additionally, the selenium dose used in the comparator group (100 μg/day) was lower than doses used in some previous CAT studies, which should be considered when interpreting the comparative effects of selenium supplementation [40,41].

## 5. Conclusions

In conclusion, thyroid-directed photobiomodulation (PBM) therapy was associated with a significant improvement in serum GSH concentrations compared with selenium plus vitamin D supplementation in treatment-naïve euthyroid women with chronic autoimmune thyroiditis. In parallel with the improvement in systemic antioxidant status, PBM was associated with reductions in thyroid autoantibody concentrations and thyroid volume, together with favorable changes in thyroid function parameters. These findings suggest that modulation of oxidative stress and improvement of redox balance may represent one potential biological pathway contributing to the observed effects of PBM in CAT.

To our knowledge, this is the first prospective clinical study to evaluate serum GSH as the primary outcome following thyroid-directed PBM administered as a standalone intervention in treatment-naïve euthyroid women with CAT. Although these findings are promising, they should be interpreted considering the non-randomized design, relatively small sample size, and single-center setting. Larger randomized controlled trials with longer follow-up and comprehensive assessment of oxidative stress, inflammatory, and immunological biomarkers are required to confirm these findings, optimize PBM dosimetry, and further define the potential role of PBM as an adjunctive intervention in autoimmune thyroid disease.

## Figures and Tables

**Table 1:** Photobiomodulation device specifications and treatment parameters.

Parameter	Specification
Laser device	Omega XP (Omega Laser Systems Ltd., Essex, UK)
Laser type	GaAlAs diode laser
Wavelength	820 nm
Emission mode	Continuous wave (CW)
Output power	200 mW
Spot size	0.125 cm^2^
Power density	1.6 W/cm^2^ (1600 mW/cm^2^)
Beam delivery	Single infra-red probe
Beam angle	90°
Number of irradiation points	8 (4 at each thyroid lobe)
Irradiation time per point	20 s
Energy per point	4 J
Total irradiation time	160 s
Fluence per point	32 J/cm^2^
Total energy per session	32 J
Total fluence/session	256 J/cm^2^
Treatment frequency	Twice weekly (excluding weekend)
Total treatment duration	3 weeks
Total number of sessions	6
Application technique	Stationary
Laser–tissue distance	In contact

**Table 2:** Baseline demographic and clinical characteristics of the study participants.

Demographic and Clinical Values	Total (N = 50) Mean ± SD or *n* (%) or Median (IQR)	PBM-Treated Group (*n* = 25) Mean ± SD or *n* (%) or Median (IQR)	Supplementation Group (*n* = 25) Mean ± SD or *n* (%) or Median (IQR)
Age	29.96 ± 4.45	30.04 ± 4.63	29.88 ± 4.35
Gender: Female	50 (100.0)	25 (100.0)	25 (100.0)
Post-diagnostic interval (months)	12.00 (4.25–24.00)	12.00 (1.00–24.00)	12.00 (5.50–24.00)
BMI (kg/m^2^)	30.62 ± 3.48	30.56 ± 3.71	30.68 ± 3.31

**Table 3:** Longitudinal changes in clinical, biochemical and thyroid-related parameters in the PBM-treated and supplementation groups.

Variable	PBM-Treated Group			Supplementation Group			Between-Group Comparison at Baseline (T0)	Within Group Analysis for PBM Treated Group	Within Group Analysis for Supplementation Group	Time × Group Interaction Effect
	T0 Mean ± SD or Median (IQR)	T1 Mean ± SD or Median (IQR)	T2 Mean ± SD or Median (IQR)	T0 Mean ± SD or Median (IQR)	T1 Mean ± SD or Median (IQR)	T2 Mean ± SD or Median (IQR)				
Waist-to-Hip ratio	0.88 ± 0.06	0.88 ± 0.06	0.86 ± 0.06	0.90 ± 0.07	0.90 ± 0.07	0.90 ± 0.07	t_(48)_ = 0.652 *p* = 0.517	F_(2,48)_ = 6.252 *p* = 0.011 η^2^p = 0.207	F_(2,48)_ = 0.590 *p* = 0.491 η^2^p = 0.024	F_(2,96)_ = 3.685 *p* = 0.038 η^2^p = 0.071
BMI (kg/m^2^)	30.56 ± 3.71	30.54 ± 3.82	29.88 ± 3.84	30.68 ± 3.31	30.84 ± 3.26	30.88 ± 3.30	t_(48)_ = 0.139 *p* = 0.890	F_(2,48)_ = 11.436 *p* < 0.0001 η^2^p = 0.323	F_(2,48)_ = 1.466 *p* = 0.240 η^2^p = 0.058	F_(2,96)_ = 10.421 *p* < 0.0001 η^2^p = 0.411
TSH (μIU/mL)	2.96 (1.55–3.80)	2.50 (1.40–2.90)	2.20 (1.20–2.60)	2.80 (1.38–3.80)	3.20 (2.10–4.20)	3.10 (2.50–4.40)	*U* = 281.000 *p* = 0.541	χ^2^_(2)_ = 26.926 *p* < 0.0001 Kendall’s W = 0.538	χ^2^_(2)_ = 7.505 *p* = 0.023 Kendall’s W = 0.150	F_(2,48)_ = 16.760 *p* < 0.0001 η^2^p = 0.411
FT4 (ng/dL)	1.42 ± 0.25	2.42 ± 1.35	2.13 ± 0.96	1.57 ± 0.39	1.36 ± 0.36	1.31 ± 0.26	t_(48)_ = 1.618 *p* = 0.112	F_(2,48)_ = 7.824 *p* = 0.001 η^2^p = 0.246	F_(2,48)_ = 4.589 *p* = 0.015 η^2^p = 0.161	F_(2,96)_ = 10.869 *p* < 0.0001 η^2^p = 0.185
FT3 (pmol/L)	3.73 ± 1.15	5.00 ± 1.34	5.08 ± 0.55	4.22 ± 1.04	3.80 ± 0.99	3.72 ± 0.93	t_(48)_ = 1.511 *p* = 0.137	F_(2,48)_ = 10.767 *p* = 0.001 η^2^p = 0.310	F_(2,48)_ = 4.178 *p* = 0.021 η^2^p = 0.148	F_(2,96)_ = 14.897 *p* < 0.0001 η^2^p = 0.237
Anti-TPO (lU/mL)	443.00 (149.55–595.85)	105.00 (69.25–175.50)	83.00 (46.50–117.50)	268.50 (38.50–554.60)	157.00 (37.60–353.50)	121.00 (47.90–443.40)	*U* = 235.500 *p* = 0.135	χ^2^_(2)_ = 38.323 *p* < 0.0001 Kendall’s W = 0.766	χ^2^_(2)_ = 3.920 *p* = 0.141 Kendall’s W = 0.078	F_(2,48)_ = 7.513 *p* = 0.001 η^2^p = 0.238
Anti-TG (lU/mL)	173.20 (25.85–420.52)	67.60 (20.00–205.00)	40.00 (17.50–109.42)	52.88 (19.31–236.80)	108.70 (18.65–340.25)	97.90 (18.90–385.60)	*U* = 237.000 *p* = 0.208	χ^2^_(2)_ = 18.589 *p* < 0.0001 Kendall’s W = 0.371	χ^2^_(2)_ = 0.320 *p* = 0.852 Kendall’s W = 006	F_(2,48.09)_ = 7.389 *p* = 0.002 η^2^p = 0.238
Thyroid volume (mL)	15.86 ± 3.16	13.56 ± 1.46	12.14 ± 3.39	15.43 ± 3.13	15.37 ± 3.37	15.43 ± 3.44	t_(48)_ = 0.485 *p* = 0.630	F_(2,48)_ = 13.646 *p* < 0.0001 η^2^p = 0.362	F_(2,48)_ = 0.121 *p* = 0.886 η^2^p = 0.005	F_(2,96)_ = 13.081 *p* < 0.0001 η^2^p = 0.214
Glutathione (mg/L)	204.00 (171.50–278.50)	255.00 (218.00–299.00)	246.00 (206.50–286.50)	230.00 (176.50–316.50)	211.00 (129.50–286.00)	210.00 (136.00–240.00)	*U* = 351.500 *p* = 0.449	χ^2^_(2)_ = 35.120 *p* < 0.0001 Kendall’s W = 0.702	χ^2^_(2)_ = 1.953 *p* = 0.377 Kendall’s W = 0.039	F_(2,48)_ = 19.101 *p* < 0.0001 η^2^p = 0.443

## Data Availability

The data presented in this study are available from Venera Berisha-Muharremi upon reasonable request. The data are not publicly available due to privacy and ethical restrictions.

## Supplementary Materials

The following supporting information can be downloaded at: https://www.mdpi.com/article/10.3390/antiox15091105/s1, Table S1: TREND (Transparent Reporting of Evaluations with Nonrandomized Designs) statement. Reference [42] is cited in the Supplementary Materials.

## Abbreviations

The following abbreviations are used in this manuscript:

CAT	Chronic Autoimmune Thyroiditis
PBM	Photobiomodulation
GSH	Serum Glutathione
TSH	Thyroid-Stimulating Hormone
FT3	Free Triiodothyronine
FT4	Free Thyroxine
Anti-TPO	Anti-thyroperoxidase Antibody
Anti-TG	Anti-thyroglobulin Antibody
LT4	Levothyroxine
HT	Hashimoto’s Thyroiditis
ROS	Reactive Oxygen Species
H_2_O_2_	Hydrogen Peroxide
DNA	Deoxyribonucleic Acid
NF-κB	Nuclear Factor Kappa
TNF-α	Tumor Necrosis Factor-Alpha
IL-1β	Interleukin-1β
IL-6	Interleukin-6
IFN-γ	Interferon-gamma
CCO	Cytochrome C Oxidase
NO	Nitric Oxide
GPx	Glutathione Peroxidase
GSSG	Oxidized Glutathione
BMI	Body mass index
WHR	Waist-to-hip ratio
SD	Standard Deviation
IQR	Interquartile Range
LMMS	Linear Mixed-Effects Models
