# Supplementary material for: Comparative Effects of Photobiomodulation Therapy Versus Conventional Antioxidant Supplementation on Serum Glutathione Levels in Euthyroid Chronic Autoimmune Thyroiditis: A Prospective Open-Label Interventional Clinical Trial
Source: Antioxidants (Basel). 2026 Sep 1;15(9):1105. doi: 10.3390/antiox15091105 (PMC13603426; doi:10.3390/antiox15091105)
Supplement: Supplementary file 1 [file antioxidants-15-01105-s001.zip › antioxidants-4477496-supplementary-Table S1.pdf]

TREND (Transparent Reporting of Evaluations with Nonrandomized Designs) statement

Topic	Item no.	Recommendations	Reported on page no.
**TITLE and ABSTRACT**			
Title and Abstract	1	• Information on how units were allocated to interventions	1,2
		• Structured abstract recommended	1,2
		• Information on target population or study sample	1,2
**INTRODUCTION**			
Background	2	• Scientific background and explanation of rationale	3-6
		• Theories used in designing behavioral interventions	3-6
**METHODS**			
Participants	3	• Eligibility criteria for participants, including criteria at different levels in recruitment/sampling plan (e.g., cities, clinics, subjects)	6-13
		• Method of recruitment (e.g., referral, self-selection), including the sampling method if a systematic sampling plan was implemented	N/A
		• Recruitment setting	6
		• Settings and locations where the data were collected	11
Interventions	4	• Details of the interventions intended for each study condition and how and when they were actually administered, specifically including:	
		o Content: what was given?	8-13
		o Delivery method: how was the content given?	8-13
		o Unit of delivery: how were subjects grouped during delivery?	8-13
		o Deliverer: who delivered the intervention?	8-13
		o Setting: where was the intervention delivered?	8,9
		o Exposure quantity and duration: how many sessions or episodes or events were intended to be delivered? How long were they intended to last?	8,9
		o Time span: how long was it intended to take to deliver the intervention to each unit?	8,9
		o Activities to increase compliance or adherence (e.g., incentives)	8,9
Objectives	5	• Specific objectives and hypotheses	6
Outcomes	6	• Clearly defined primary and secondary outcome measures	9-11
		• Methods used to collect data and any methods used to enhance the quality of measurements	9-11
		• Information on validated instruments such as psychometric and biometric properties	9-11
Sample size	7	• How sample size was determined and, when applicable, explanation of any interim analyses and stopping rules	12
Assignment method	8	• Unit of assignment (the unit being assigned to study condition, e.g., individual, group, community)	9-11
		• Method used to assign units to study conditions, including details of any restriction (e.g., blocking, stratification, minimization)	N/A
		• Inclusion of aspects employed to help minimize potential bias induced due to non-randomization (e.g., matching)	12
Blinding (masking)	9	• Whether or not participants, those administering the interventions, and those assessing the outcomes were blinded to study condition assignment; if so, statement regarding how the blinding was accomplished and how it was assessed	N/A
Unit of Analysis	10	• Description of the smallest unit that is being analysed to assess intervention effects (e.g., individual, group, or community)	N/A
		• If the unit of analysis differs from the unit of assignment, the analytical method used to account for this (e.g., adjusting the standard error	N/A

		estimates by the design effect or using multilevel analysis)	
Statistical methods	11	• Statistical methods used to compare study groups for primary methods outcome(s), including complex methods for correlated data	11
		• Statistical methods used for additional analyses, such as subgroup analyses and adjusted analysis	11
		• Methods for imputing missing data, if used	11
		• Statistical software or programs used	11
**RESULTS**			
Participant flow	12	• Flow of participants through each stage of the study: enrollment, assignment, allocation and intervention exposure, follow-up, analysis (a diagram is strongly recommended)	12
		○ Enrollment: the numbers of participants screened for eligibility, found to be eligible or not eligible, declined to be enrolled, and enrolled in the study	12
		○ Assignment: the numbers of participants assigned to a study condition	9-11
		○ Allocation and intervention exposure: the number of participants assigned to each study condition and the number of participants who received each intervention	9-11
		○ Follow-up: the number of participants who completed the follow-up or did not complete the follow-up (i.e., lost to follow-up), by study condition	9-11
		○ Analysis: the number of participants included in or excluded from the main analysis, by study condition	12
		• Description of protocol deviations from study as planned, along with reasons	9-11
Recruitment	13	• Dates defining the periods of recruitment and follow-up	9-11
Baseline data	14	• Baseline demographic and clinical characteristics of participants in each study condition	13-21
		• Baseline characteristics for each study condition relevant to specific disease prevention research	13-21
		• Baseline comparisons of those lost to follow-up and those retained, overall and by study condition	13-21
		• Comparison between study population at baseline and target population of interest	13-21
Baseline equivalence	15	• Data on study group equivalence at baseline and statistical methods used to control for baseline differences	13-21
Numbers analyzed	16	• Number of participants (denominator) included in each analysis for each study condition, particularly when the denominators change for different outcomes; statement of the results in absolute numbers when feasible	13-21
		• Indication of whether the analysis strategy was "intention to treat" or, if not, description of how non-compliers were treated in the analyses	13-21
Outcomes and estimation	17	• For each primary and secondary outcome, a summary of results for each estimation study condition, and the estimated effect size and a confidence interval to indicate the precision	13-21
		• Inclusion of null and negative findings	N/A
		• Inclusion of results from testing pre-specified causal pathways through which the intervention was intended to operate, if any	N/A
Ancillary analyses	18	• Summary of other analyses performed, including subgroup or restricted analyses, indicating which are pre-specified or exploratory	N/A
Adverse events	19	• Summary of all important adverse events or unintended effects in each study condition (including summary measures, effect size estimates, and confidence intervals)	N/A
**DISCUSSION**			
Interpretation	20	• Interpretation of the results, taking into account study hypotheses, sources	13-26

		of potential bias, imprecision of measures, multiplicative analyses, and other limitations or weaknesses of the study	
		• Discussion of results taking into account the mechanism by which the intervention was intended to work (causal pathways) or alternative mechanisms or explanations	13-19
		• Discussion of the success of and barriers to implementing the intervention, fidelity of implementation	13-18
		• Discussion of research, programmatic, or policy implications	13-19
Generalizability	21	• Generalizability (external validity) of the trial findings, taking into account the study population, the characteristics of the intervention, length of follow-up, incentives, compliance rates, specific sites/settings involved in the study, and other contextual issues	N/A
Overall evidence	22	• General interpretation of the results in the context of current evidence and current theory	13-18

Developed from: Des Jarlais, D. C., Lyles, C., Crepaz, N., & the Trend Group (2004) Improving the reporting quality of nonrandomized evaluations of behavioral and public health interventions: The TREND statement. American Journal of Public Health, 94, 361-366.
